# Efficacy of self-monitored blood pressure, with or without telemonitoring, for titration of antihypertensive medication (TASMINH4): an unmasked randomised controlled trial

**DOI:** 10.1016/S0140-6736(18)30309-X

**Published:** 2018-03-10

**Authors:** Richard J McManus, Jonathan Mant, Marloes Franssen, Alecia Nickless, Claire Schwartz, James Hodgkinson, Peter Bradburn, Andrew Farmer, Sabrina Grant, Sheila M Greenfield, Carl Heneghan, Susan Jowett, Una Martin, Siobhan Milner, Mark Monahan, Sam Mort, Emma Ogburn, Rafael Perera-Salazar, Syed Ahmar Shah, Ly-Mee Yu, Lionel Tarassenko, F D Richard Hobbs, TASMINH4 investigators, Brendan Bradley, Chris Lovekin, David Judge, Luis Castello, Maureen Dawson, Rebecca Brice, Bethany Dunbabin, Sophie Maslen, Heather Rutter, Mary Norris, Lauren French, Michael Loynd, Pippa Whitbread, Luisa Saldana Ortaga, Irene Noel, Karen Madronal, Julie Timmins, Peter Bradburn, Lucy Hughes, Beth Hinks, Sheila Bailey, Sue Read, Andrea Weston, Somi Spannuth, Sue Maiden, Makiko Chermahini, Ann McDonald, Shelina Rajan, Sue Allen, Brenda Deboys, Kim Fell, Jenny Johnson, Helen Jung, Rachel Lister, Ruth Osborne, Amy Secker, Irene Qasim, Kirsty William, Abi Harris, Susan Zhao, Elaine Butcher, Pauline Darbyshire, Sarah Joshi, Jon Davies, Claire Talbot, Eleanor Hoverd, Linda Field, Tracey Adcock, Julia Rooney, Nina Cooter, Aaron Butler, Naomi Allen, Maria Abdul-Wahab, Kathryn McNicholas, Lara Peniket, Kate Dodd, Julie Mugurza, Richard Baskerville, Rakshan Syed, Clare Bailey, Jill Adams, Paul Uglow, Neil Townsend, Alison Macleod, Charlotte Hawkins, Suparna Behura, Jonathan Crawshaw, Robin Fox, Waleed Doski, Martin Aylward, Christine A'Court, David Rapley, Jo Walsh, Paul Batra, Ana Seoane, Sluti Mukherjee, Jonathan Dixon, Peter Arthur, Karen Sutcliffe, Costas Paschallides, Richard Woof, Peter Winfrey, Matthew Clark, Roya Kamali, Paul Thomas, David Ebbs, Liz Mather, Andre Beattie, Karim Ladha, Larisa Smondulak, Surinder Jemahl, Peter Hickson, Liam Stevens, Tony Crockett, David Shukla, Ian Binnian, Paul Vinson, Nigel DeKare-Silver, Ramila Patel, Ivor Singh, Louise Lumley, Glennis Williams, Mark Webb, Jack Bambrough, Neetul Shah, Hergeven Dosanjh, Frank Spannuth, Carolyn Paul, Jude Ganesegaram, Laurie Pike, Vijaysundari Maheswaran, Farah Paruk, Stephen Ford, Vineeta Verma, Kate Milne, Farhana Lockhat, Jennifer Ferguson, Anne-Marie Quirk, Hugo Wilson, David Copping, Sam Bajallan, Simria Tanvir, Faheem Khan, Tom Alderson, Amar Ali, Richard Young, Umesh Chauhan, Lindsey Crockett, Louise McGovern, Claire Cubitt, Simon Weatherill, Abdul Tabassum, Philip Saunders, Naresh Chauhan, Samantha Johnson, Jo Walsh, Inderjit Marok, Rajiv Sharma, William Lumb, John Tweedale, Ian Smith, Lawrence Miller, Tanveer Ahmed, Mark Sanderson, Claire Jones, Peter Stokell, Matthew J Edwards, Andrew Askey, Jason Spencer, Kathryn Morgan, Kyle Knox, Robert Baker, Crispin Fisher, Rachel Halstead, Neil Modha, David Buckley, Catherine Stokell, John Gerald McCabe, Jennifer Taylor, Helen Nutbeam, Richard Smith, Christopher MacGregor, Sam Davies, Mark Lindsey, Simon Cartwright, Jonathan Whittle, Julie Colclough, Alison Crumbie, Nicholas Thomas, Vattakkatt Premchand, Rafia Hamid, Zishan Ali, John Ward, Philip Pinney, Stephen Thurston, Tina Banerjee

**Affiliations:** a Nuffield Department of Primary Care Health Sciences, University of Oxford, Oxford, UK; b Institute of Biomedical Engineering, University of Oxford, Oxford, UK; c Primary Care Unit, Department of Public Health and Primary Care, University of Cambridge, Cambridge, UK; d Institute of Applied Health Research, University of Birmingham, Birmingham, UK; e Musculoskeletal Research Unit, Translational Health Sciences, Bristol Medical School, University of Bristol, Southmead Hospital, Southmead, Bristol, UK

## Abstract

**Background:**

Studies evaluating titration of antihypertensive medication using self-monitoring give contradictory findings and the precise place of telemonitoring over self-monitoring alone is unclear. The TASMINH4 trial aimed to assess the efficacy of self-monitored blood pressure, with or without telemonitoring, for antihypertensive titration in primary care, compared with usual care.

**Methods:**

This study was a parallel randomised controlled trial done in 142 general practices in the UK, and included hypertensive patients older than 35 years, with blood pressure higher than 140/90 mm Hg, who were willing to self-monitor their blood pressure. Patients were randomly assigned (1:1:1) to self-monitoring blood pressure (self-montoring group), to self-monitoring blood pressure with telemonitoring (telemonitoring group), or to usual care (clinic blood pressure; usual care group). Randomisation was by a secure web-based system. Neither participants nor investigators were masked to group assignment. The primary outcome was clinic measured systolic blood pressure at 12 months from randomisation. Primary analysis was of available cases. The trial is registered with ISRCTN, number ISRCTN 83571366.

**Findings:**

1182 participants were randomly assigned to the self-monitoring group (n=395), the telemonitoring group (n=393), or the usual care group (n=394), of whom 1003 (85%) were included in the primary analysis. After 12 months, systolic blood pressure was lower in both intervention groups compared with usual care (self-monitoring, 137·0 [SD 16·7] mm Hg and telemonitoring, 136·0 [16·1] mm Hg *vs* usual care, 140·4 [16·5]; adjusted mean differences *vs* usual care: self-monitoring alone, −3·5 mm Hg [95% CI −5·8 to −1·2]; telemonitoring, −4·7 mm Hg [–7·0 to −2·4]). No difference between the self-monitoring and telemonitoring groups was recorded (adjusted mean difference −1·2 mm Hg [95% CI −3·5 to 1·2]). Results were similar in sensitivity analyses including multiple imputation. Adverse events were similar between all three groups.

**Interpretation:**

Self-monitoring, with or without telemonitoring, when used by general practitioners to titrate antihypertensive medication in individuals with poorly controlled blood pressure, leads to significantly lower blood pressure than titration guided by clinic readings. With most general practitioners and many patients using self-monitoring, it could become the cornerstone of hypertension management in primary care.

**Funding:**

National Institute for Health Research via Programme Grant for Applied Health Research (RP-PG-1209-10051), Professorship to RJM (NIHR-RP-R2-12-015), Oxford Collaboration for Leadership in Applied Health Research and Care, and Omron Healthcare UK.

## Introduction

Hypertension is a leading risk factor for cardiovascular disease, the greatest cause of morbidity and mortality internationally.^1^, ^2^ Despite the widespread availability of effective treatment, control of hypertension in the community remains sub-optimal.^3^, ^4^ Key reasons for this include clinical inertia, poor adherence, and organisational failure.^5^, ^6^, ^7^

Self-monitoring as part of a self-management strategy is an effective way to improve blood pressure control, but is only applicable to those willing to self-titrate.^8^, ^9^ Self-monitoring in isolation is not associated with better blood pressure control, but is effective in combination with other co-interventions.^10^ Many primary-care physicians incorporate self-monitored readings in their treatment decisions, but there is considerable variation in practice,^11^ and mixed evidence to support such an approach: two previous European studies with 12 months follow-up where physicians used self-monitored blood pressure to explicitly titrate antihypertensive medication have resulted in worse blood pressure control.^12^, ^13^ In both studies, the prescribing physicians were masked to the method of blood pressure measurement and used a common target blood pressure for both home and clinic readings (140/90 mm Hg) as opposed to lower home targets (typically 135/85 mm Hg) as recommended by contemporary guidelines.^14^ An intervention including telemonitoring and self-monitoring with general practitioner (GP) titration of antihypertensives in Scotland showed significant reductions in blood pressure using lower home targets (home 135/85 mm Hg *vs* clinic 140/90 mm Hg) but only followed up patients for 6 months.^15^

**Research in context**

**Evidence before this study**

We updated our systematic reviews from inception to Jan 2, 2018, in MEDLINE, Embase, and the Cochrane Library with search terms designed to capture all trials using self-monitoring of blood pressure, with or without telemonitoring, to guide the titration of antihypertensive treatment without other co-interventions. Search terms included ambulatory blood pressure monitoring, home or self monitoring, telemedicine, and randomised controlled trials, and we had no language restrictions. We found three trials that fulfilled these criteria, one of which used telemonitoring. Two trials (the Treatment of Hypertension Based on Home or Office Blood Pressure trial and the Home Versus Office Measurement, Reduction of Unnecessary Treatment Study) found that when clinicians used home readings to titrate treatment, this led to worse blood pressure control and less treatment after 1 year as compared with using clinic readings. Identical treatment targets were used for both home and clinic blood pressures. A third trial (Health Impact of nurse-led Telemetry Services) used a telemonitoring-based service to capture home readings and guide treatment compared with usual care, both using guideline recommended targets which were lower for home readings. The telemonitoring, home titration group had lower blood pressure than clinic-based care after 6 months.

**Added value of this study**

To the best of our knowledge, this is the first trial in primary care of antihypertensive titration using self-monitored blood pressure, with or without telemonitoring, to show a benefit in terms of blood pressure after 12 months. Differences in blood pressure recorded at 6 months were amplified by 1 year suggesting that the intervention increased in efficacy in the second 6 months. This was achieved without increased workload and using internationally recommended targets for home and clinic blood pressure, the former lower by 5/5 mm Hg. Additional benefit from telemonitoring was seen in terms of lower blood pressure after 6 months suggesting more efficient titration.

**Implications of all the available evidence**

This study provides good evidence that self-monitoring can be used, with or without telemonitoring, to guide antihypertensive titration in a primary-care setting for people with poorly controlled blood pressure, provided that lower targets are used for home measurements. The decision to use telemonitoring will depend on whether the additional benefits in terms of speed of titration and ease of use are considered worthwhile. The reductions in blood pressure observed would be expected to reduce stroke risk by around 20% and coronary heart disease risk by about 10%. Self-monitoring can be recommended for the ongoing management of hypertension in primary care in all patients who wish to use it, and general practitioners should incorporate self-monitored readings into their titration of blood pressure medications.

Current UK hypertension guidelines reflect the uncertainty in the literature by recommending self-monitoring of blood pressure as one option for the diagnosis of hypertension, but only for longer term management in the context of white coat hypertension.^14^ They gave an explicit research recommendation that a new trial was needed to understand the place of self-monitoring and telemonitoring of blood pressure in the management of hypertension in primary care. The TASMINH4 trial aimed to evaluate whether GPs using self-monitored blood pressure to titrate antihypertensive medication in people with treated but inadequately controlled hypertension, resulted in lower systolic blood pressure than usual care and whether telemonitoring resulted in lower blood pressure than self-monitoring alone.

## Methods

### Study design and participants

This was an unmasked randomised controlled trial with automated ascertainment of the end point. Detailed methods have been published previously.^16^

Potentially eligible participants were identified using automated searches of electronic primary care patient records in practices in England, UK. The searches identified individuals potentially eligible in terms of age, hypertension diagnosis, current medication, and last recorded systolic blood pressure above 145 mm Hg. Inclusion criteria were age older than 35 years, with a diagnosis of hypertension, taking no more than three antihypertensive agents, but with clinic blood pressure not controlled below 140/90 mm Hg. They had to be on stable antihypertensive medication for at least 4 weeks before randomisation and free from orthostatic hypotension, atrial fibrillation, dementia, or chronic kidney disease of grade 4 or worse, or chronic kidney disease with proteinuria.

A trial steering group and data monitoring committee supervised the trial. Ethical approval was gained from Oxford NHS Research Ethics Committee B (14/SC/0218). All patients gave written informed consent.

### Randomisation and masking

Potentially eligible patients were invited to research clinics held in their own general practices between Nov 13, 2014, and Feb 3, 2016 where they were screened for eligibility, informed consent taken, baseline measurements taken, and questionnaires administered. Eligible and willing participants were randomly assigned (1:1:1), using a secure web-based system, to GP antihypertensive titration using clinic readings (usual care group), using self-monitoring alone (self-monitoring group), or using self-monitoring with telemonitoring (telemonitoring group), with stratification by practice and minimisation on baseline blood pressure, sex, and blood pressure target.

Neither participants nor investigators were masked to group assignment in this open trial. Outcome measurement was not masked but used the automatic mode of the sphygmomanometer to measure blood pressure without the need for intervention by the investigator other than to place the cuff and switch the device on.

### Procedures

Following randomisation, all participants were asked to attend their own GP for a medication review. Participants randomly assigned to usual care were thereafter managed with titration of antihypertensive treatment based on clinic blood pressure measurements at the discretion of their attending health-care professional.

Participants randomly assigned to self-monitoring were taught to use a validated automated electronic sphygmomanometer (Omron M10-IT; Omron Healthcare Europe, Hoofddorp, Netherlands).^17^ They were asked to monitor their own blood pressure in their non-dominant arm, twice each morning and evening, for the first week of every month using standard recommendations and their GPs were asked to use the self-monitored measurements for titration of antihypertensive medication.^14^, ^18^

For those self-monitoring alone, a simple colour chart was used to train participants to attend their practice for blood pressure checks in the light of very high or very low readings. At the end of each monitoring week they were asked to record their readings on paper and send them for review to their practice in a reply-paid envelope. Participants in the telemonitoring group were trained to send readings via a simple free SMS text-based telemonitoring service with web-based data entry back-up. The telemonitoring system incorporated an algorithm that alerted participants to contact their surgery in the light of very high or very low readings, reminded them if insufficient readings were transmitted, prompted them to make contact with their practice if their average blood pressure was above target, and presented readings to attending clinicians via a web interface (appendix p 9). This secure web page automatically calculated mean blood pressure for each monitoring week, highlighted very high or very low readings, and presented a graphical display of blood pressure measurements.

Attending clinicians were asked to review both self-monitoring and telemonitoring groups' readings on a monthly basis and usual care patients as often as they wished. Blood pressure targets were based on current National Guidelines adjusted for self-monitoring: lower than 140/90 mm Hg (<135/85 mm Hg at home) for those younger than 80 years, lower than 150/90 mm Hg (<145/85 mm Hg at home) for those 80 years or older, and lower than 140/80 mm Hg (<135/75 mm Hg at home) for those with diabetes.^14^ Clinicians in the trial had complete freedom to adjust antihypertensive and other medication as they sought fit, regardless of which group an individual was randomly assigned to and with no restriction on type of drug used. All participants were followed up at 6 and 12 months by research nurses.

### Outcomes

The primary outcome was clinic measured systolic blood pressure, adjusted for baseline covariates at 12 months. Blood pressure was measured by a research nurse using a validated monitor, six times at baseline and each follow-up appointment in a standardised fashion, using the same arm and cuff size each time, in a seated position after at least 5 min rest.^19^ The mean of the second and third readings was used in the primary outcome.

Other outcomes included other measures of blood pressure at 6 and 12 months (systolic and diastolic, in each case mean of the second and third readings and the mean of the second to the sixth readings), adverse events (side-effects, cardiovascular events, anxiety),^20^, ^21^ medication prescription (number and defined daily dose),^22^ self-reported adherence (Medication Adherence Rating Scale [MARS]),^23^ weight and waist circumference, lifestyle factors (alcohol, diet, exercise, and smoking),^24^ and quality of life (EQ-5D-5L).^25^ Cost-effectiveness and qualitative sub-studies will be reported separately.

### Statistical analysis

It was estimated that 1110 patients (370 per group, allowing for 15% attrition) would be required to detect a 5 mm Hg systolic blood pressure difference between the groups with 90% power and an adjusted alpha of 0·017 (two-sided) to account for all three pairwise comparisons. This calculation was based on an assumption of a common SD of 17 mm Hg and a three-way pairwise comparison.

Analysis was done following database lock on an intention-to-treat basis fitting a linear mixed-effects model, which modelled outcome data collected at 6 and 12 months from randomisation, adjusting for baseline blood pressure measure. Practices and measurements from the same participant were accounted for by means of random intercepts. Time (categorical measure) and randomised group were fitted as fixed effects, with minimisation variables (sex and blood pressure target), and history of cardiovascular disease fitted as covariates. An interaction term between time and randomisation group was included so that the treatment effect could be assessed at each timepoint. The self-monitoring and telemonitoring groups were first compared with usual care and then with each other since both were found more effective than usual care.^16^

The continuous secondary outcomes were analysed by means of a linear mixed-effects model using the same strategy as for the primary outcome. Binary outcomes for symptoms were analysed by means of a logistic mixed-effects model and group differences presented as adjusted odds ratios (ORs) with 95% CIs. Random effects were used to account for repeated measurements and practice. Smoking was analysed using a log-binomial model and group differences presented as adjusted relative risks (RRs), accounting for baseline smoking, with 95% CIs.

Sensitivity analyses examined the robustness of the results using different approaches to obtaining mean blood pressure, replacing missing values with other available blood pressure measures, or through multiple imputation.^16^ A statistical test of interaction was done to assess whether the effect of the interventions was consistent across the prespecified subgroups: age, sex, blood pressure target, baseline blood pressure, Index of Multiple Deprivation (IMD), and history of cardiovascular disease. A detailed statistical analysis plan was prepared before the final analysis and unmasking of treatment allocation (appendix pp 11–26).

Two amendments to the protocol were approved during the trial: first to clarify the safety reporting requirements and second to clarify that National Institute for Health and Care Excellence (NICE) guidance for self-monitoring of blood pressure, which had been operationalised from the start of the trial, was being followed in both intervention groups. Analysis was done using STATA version 14.2. The trial is registered with ISRCTN, number ISRCTN 83571366.

### Role of the funding source

The funders and sponsors of the study had no role in study design, data collection, data analysis, data interpretation, writing of the report, or in the decision to submit for publication. The corresponding author (RJM) together with LMY, AN, and SM had full access to all the data in the study. RJM had final responsibility for the decision to submit for publication.

## Results

**Figure 1:**
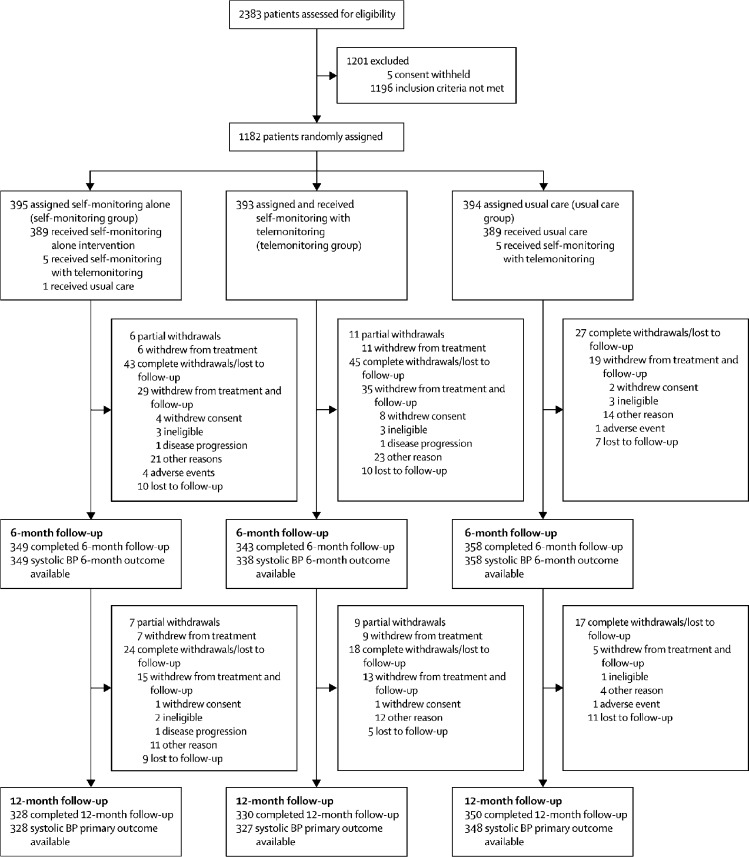
Trial profile Detail on reasons for exclusion are in the appendix (p 5). Some individuals attended 12 months and not 6 months follow-up and vice versa. BP=blood pressure.

Of 2383 individuals assessed for eligibility in 142 practices, 1201 (50%) were excluded: 1196 (50%) did not meet the inclusion criteria and five (<1%) withheld their consent (figure 1). The main reasons for exclusion were blood pressure controlled (blood pressure <140/90 mm Hg, 1048 patients), orthostatic hypertension (86 patients), or did not have a stable dose of antihypertensive medication (22 patients; appendix p 5). The remaining 1182 (50%) patients from 138 practices were enrolled and randomly assigned: 394 (33%) to usual care, 395 (33%) to self-monitoring alone, and 393 (33%) to self-monitoring with telemonitoring. Early on in the trial, ten (<1%) patients randomly assigned to either self-monitoring alone or usual care briefly received the telemonitoring intervention due to a misunderstanding. Nine (<1%) participants who were randomised withdrew and removed consent for their data to be used; they were not included in any analysis.

The groups were well matched (table 1): mean age was 66·9 years (SD 9·4), just over half were male, mean systolic blood pressure was 153·1/85·5 (SD 14·0/10·3) mm Hg, and mean time since hypertension diagnosis was 10·2 (SD 8·4) years.

**Table 1:** Baseline characteristics

		**Usual care group (n=393)**	**Self-monitoring group (n=391)**	**Telemonitoring group (n=389)**
Age (years)		66·8 (9·4)	67·0 (9·6)	67·0 (9·3)
Systolic blood pressure (mm Hg)		153·1 (14·0)	152·9 (13·6)	153·2 (14·3)
Diastolic blood pressure (mm Hg)		86·0 (10·3)	85·1 (10·5)	85·5 (10·0)
Sex				
	Female	183 (47%)	181 (46%)	181 (47%)
	Male	210 (53%)	210 (54%)	208 (53%)
Ethnicity				
	White	384 (98%)	373 (95%)	370 (95%)
	Black	6 (2%)	8 (2%)	6 (2%)
	Asian	3 (1%)	7 (2%)	6 (2%)
	Mixed	0	2 (1%)	5 (1%)
	Other, missing	0	1 (<1%)	2 (1%)
Marital status				
	Married or cohabiting	305 (78%)	306 (78%)	294 (76%)
	Single, divorced, or widowed	88 (22%)	85 (22%)	95 (24%)
Occupation				
	Working full-time	79 (20%)	81 (21%)	74 (19%)
	Working part-time	38 (10%)	39 (10%)	45 (12%)
	Retired	255 (65%)	247 (63%)	240 (62%)
	Other	21 (5%)	24 (6%)	30 (8%)
Duration of hypertension (years)		10·2 (8·2)	9·8 (8·2)	10·6 (8·8)
Past medical history				
	Chronic kidney disease	27 (7%)	27 (7%)	23 (6%)
	Myocardial Infarction	8 (2%)	9 (2%)	8 (2%)
	Coronary artery bypass graft, angioplasty, or stent	14 (4%)	16 (4%)	10 (3%)
	Stroke	8 (2%)	9 (2%)	15 (4%)
	Diabetes	35 (9%)	39 (10%)	34 (9%)
	Other ongoing medical problem	238 (61%)	228 (58%)	246 (63%)
Body-mass index (kg/m^2^)		29·8 (5·8)	29·6 (8·7)	29·3 (5·3)
Waist circumference (cm)		100·7 (14·5)	99·7 (13·7)	100·1 (13·5)
Baseline number of antihypertensive medications		1·3 (0·8)	1·4 (0·8)	1·4 (0·8)
Current smoker		17 (4%)	21 (5%)	28 (7%)

Data are mean (SD) or n (%). Where relevant, some percentages might not add to 100% due to rounding.

Primary outcome data were available from 1003 (85%) participants and retention was not significantly lower in the intervention groups (328 [83%] in the self-monitoring group and 327 [83%] in the telemonitoring group *vs* 348 [88%] in the usual care group, p=0·1119 for comparison; figure 1). After 12 months, mean systolic blood pressure (measured independently in a clinic setting) was lower in both intervention groups: self-monitoring (137·0 [SD 16·7] mm Hg) and telemonitoring (136·0 [16·1] mm Hg) compared with clinic monitoring (140·4 [16·5] mm Hg): self-monitoring, adjusted mean difference (AMD) −3·5 mm Hg (95% CI −5·8 to −1·2), p=0·0029; and telemonitoring, −4·7 mm Hg (−7·0 to −2·4), p<0·0001 (table 2). There was no significant difference between the self-monitoring and telemonitoring groups (AMD −1·2 mm Hg [95% CI −3·5 to 1·2], p=0·3219).

**Table 2:** Mean blood pressure at baseline, 6 months, and 12 months for each group

	**Baseline**	**6 months**	**12 months**	6-month adjusted mean difference (95% CI, p value*) *vs* usual care	12-month adjusted mean difference (95% CI, p value*) *vs* usual care
**Systolic blood pressure (mm Hg)**					
Telemonitoring group	153·2 (14·3); n=389	139·0 (16·8); n=338	136·0 (16·1); n=327	−3·7 (−5·9 to −1·5), p=0·0012	−4·7 (−7·0 to −2·4), p<0·0001
Self-monitoring group	152·9 (13·6); n=391	140·4 (15·7); n=349	137·0 (16·7); n=328	−2·1 (−4·3 to 0·1), p=0·0584	−3·5 (−5·8 to −1·2), p=0·0029
Usual care group	153·1 (14·0); n=393	142·5 (15·4); n=358	140·4 (16·5); n=348	..	..
**Diastolic blood pressure (mm Hg)**					
Telemonitoring group	85·5 (10·0); n=389	79·8 (9·9); n=338	78·7 (9·7); n=328	−1·2 (−2·4 to −0·01), p=0·0482	−1·3 (−2·5 to −0·02), p=0·0482
Self-monitoring group	85·1 (10·5); n=391	80·3 (10·7); n=349	77·8 (10·1); n=328	−0·1 (−1·3 to 1·07), p=0·8421	−1·5 (−2·7 to −0·2), p=0·0209
Usual care group	86·0 (10·3); n=393	81·1 (10·9); n=358	79·9 (10·7); n=348	..	..

Data are mean (SD), unless otherwise stated.

* Significant at p<0·017.

Considering the sensitivity analyses, similar results were recorded when the mean of the second to the sixth readings was used, when those with one or other of the second or third measurements missing were included, where blood pressure measurements identified as outliers (ie, probably erroneous) were excluded, and where multiple imputation was used to replace missing values (appendix p 6).

After 6 months, mean systolic blood pressure in the group titrated using telemonitoring (139·0 [SD 16·8] mm Hg) but not the self-monitoring group (140·4 [15·7] mm Hg) was significantly lower than usual care (142·5 mm Hg [15·4]): telemonitoring, AMD −3·7 mm Hg (95% CI −5·9 to −1·5), p=0·0012; and self-monitoring, −2·1 mm Hg (−4·3 to 0·1), p=0·0584 (table 2). There was no significant difference between the two intervention groups (AMD −1·5 [95% CI −3·8 to 0·7], p=0·1771). No evidence of differences in diastolic blood pressure between groups was found at 6 or 12 months (table 2).

**Figure 2:**
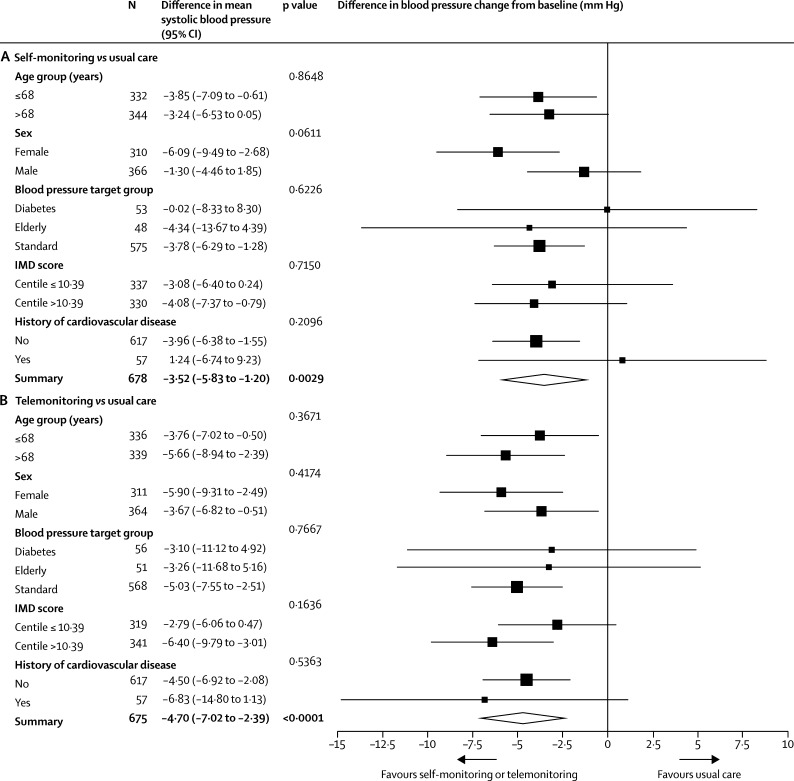
Forest plot of subgroup analyses for the change in systolic blood pressure from baseline to 12 months (A) Self-monitoring versus usual care and (B) telemonitoring versus usual care. Target blood pressures: diabetes <140/80 mm Hg (<135/75 mm Hg at home); elderly (≥80 years) <150/90 mm Hg (<145/85 mm Hg at home); and standard (all others) <140/90 mm Hg (<135/85 mm Hg at home). IMD=Index of Multiple Deprivation.

There was no evidence of an effect of prespecified subgroups on the difference between treatment groups. Some of the comparisons had wide CIs reflecting relatively few individuals in some of the groups (figure 2A, B).

After 12 months, individuals for whom self-monitoring and telemonitoring was used to titrate their antihypertensives were prescribed additional medications compared with usual care (self-monitoring 1·63 [SD 0·89], telemonitoring 1·70 [0·88], usual care 1·55 [0·85] antihypertensives): an AMD of 0·11 (95% CI 0·02 to 0·19) more medications in the self-monitoring group (p=0·0129) and 0·13 (0·04 to 0·21) more medications in the telemonitoring group (p=0·0038), both compared with usual care (table 3). Defined daily doses (DDDs)^22^ were significantly increased at 12 months in those where telemonitoring was used (DDD 2·69 [SD 1·82], AMD 0·31 [95% CI 0·15 to 0·47], p<0·0001) but not in those self-monitoring (DDD 2·42 [1·75], AMD 0·19 [0·03 to 0·36], p=0·0175), both compared with usual care (DDD 2·27 [1·65]), and this was largely driven by an increase in angiotensin-converting-enzyme inhibitors and angiotensin receptor blockers (table 3).

**Table 3:** Prescription of antihypertensive medication

	**Usual care group**		**Self-monitoring group**		**Telemonitoring group**		Self-monitoring *vs* usual care*(mean difference [95% CI], p value†**)**	Telemonitoring *vs* usual care*(mean difference [95% CI], p value†**)**	Telemonitoring *vs* self-monitoring*(mean difference [95% CI], p value†**)**
	Baseline	12 months	Baseline	12 months	Baseline	12 months			
Number of antihypertensive drugs	1·33 (0·75)	1·55 (0·85)	1·36 (0·80)	1·63 (0·89)	1·39 (0·82)	1·70 (0·88)	0·11 (0·02 to 0·19), p=0·0129	0·13 (0·04 to 0·21), p=0·0038	0·02 (−0·07 to 0·10), p=0·6808
Overall DDD	1·92 (1·48)	2·27 (1·65)	1·97 (1·57)	2·42 (1·75)	2·09 (1·65)	2·69 (1·82)	0·19 (0·03 to 0·36), p=0·0175	0·31 (0·15 to 0·47), p<0·0001	0·11 (−0·05 to 0·27), p=0·1795
DDD of thiazide and related diuretics	0·18 (0·38)	0·19 (0·39)	0·19 (0·39)	0·25 (0·42)	0·21 (0·40)	0·25 (0·44)	0·04 (0·00 to 0·08), p=0·0396	0·03 (−0·01 to 0·07), p=0·1437	−0·01 (−0·05 to 0·03), p=0·5597
DDD of beta-blockers	0·06 (0·20)	0·07 (0·21)	0·09 (0·23)	0·08 (0·23)	0·07 (0·21)	0·09 (0·23)	0·01 (−0·01 to 0·03), p=0·4157	0·01 (−0·01 to 0·03), p=0·1998	0·00 (−0·01 to 0·02), p=0·6433
DDD of angiotensin-converting-enzyme inhibitors and angiotensin II blockers	1·17 (1·28)	1·31 (1·37)	1·21 (1·36)	1·40 (1·42)	1·25 (1·40)	1·57 (1·51)	0·10 (−0·02 to 0·23), p=0·0981	0·20 (0·07 to 0·32), p=0·0018	0·09 (−0·03 to 0·22), p=0·1452
DDD of calcium antagonists	0·45 (0·69)	0·61 (0·75)	0·43 (0·69)	0·62 (0·77)	0·47 (0·69)	0·71 (0·81)	0·04 (−0·04 to 0·11), p=0·3540	0·09 (0·01 to 0·17), p=0·0247	0·05 (−0·03 to 0·13), p=0·1923
DDD of alpha 1 blockers	0·05 (0·24)	0·07 (0·32)	0·03 (0·22)	0·05 (0·25)	0·07 (0·37)	0·06 (0·33)	0·00 (−0·03 to 0·03), p=0·9112	−0·02 (−0·05 to 0·01), p=0·3136	−0·01 (−0·04 to 0·02), p=0·3772

Data are mean (SD), unless otherwise stated. DDD=defined daily dose (as classified by WHO^22^).

* Linear mixed-effect model of the outcome score at 6 and 12 months modelled against randomised group, time of visit, and its interaction with randomised group, baseline outcome score, baseline systolic blood pressure, sex, history of cardiovascular disease, target blood pressure as fixed effects, and practice as a random effect.

† Significant at p<0·017.

No difference in self-reported adherence was recorded at 12 months between the three groups. MARS questionnaire scores were 23·8 (SD 1·9) in the self-monitoring group, 24·0 (1·5) in the telemonitoring group, and 23·9 (1·9) in the usual care group (self-monitoring *vs* usual care, AMD −0·05 [95% CI −0·27 to 0·17], p=0·6619; telemonitoring *vs* usual care, AMD 0·02 [–0·20 to 0·25], p=0·8334).^23^

Reported potential side-effects were similar between the three groups (table 4). There was no difference in anxiety between any of the groups (appendix p 7). Cardiovascular events (new atrial fibrillation, angina, myocardial infarction, coronary artery bypass graft or angioplasty, stroke, peripheral vascular disease, or heart failure) were recorded in nine patients in the usual care group, 12 in the self-monitoring group, and 11 in the telemonitoring group.

**Table 4:** Participants who reported a hypertension medication-specific symptom or adverse effect at 12 month follow-up visit (ten most reported symptoms plus hypertension-specific symptoms not reported in the top ten)

		Usual care (n=350*)	Self-monitoring group (n=328*)	Telemonitoring group (n=330*)	**Self-monitoring *vs* usual care**		**Telemonitoring *vs* usual care**		**Telemonitoring *vs* self-monitoring**	
					Adjusted odds ratio (95% CI)	p value†	Adjusted odds ratio (95% CI)	p value†	Adjusted odds ratio (95% CI)	p value†
Pain		156/348 (45%)	139/326 (43%)	144/329 (44%)	0·82 (0·52 to 1·28)	0·3816	0·80 (0·51 to 1·25)	0·3328	0·98 (0·62 to 1·55)	0·9251
Stiff joints		152/348 (44%)	146/325 (45%)	134/329 (41%)	1·02 (0·62 to 1·68)	0·9381	0·67 (0·40 to 1·10)	0·1161	0·68 (0·41 to 1·14)	0·1414
Sleep difficulties		140/349 (40%)	129/325 (40%)	137/325 (42%)	0·99 (0·58 to 1·69)	0·9747	0·89 (0·52 to 1·53)	0·6797	0·90 (0·52 to 1·56)	0·7068
Fatigue		132/348 (38%)	123/324 (38%)	122/327 (37%)	0·93 (0·56 to 1·55)	0·7851	0·76 (0·45 to 1·27)	0·2949	0·81 (0·48 to 1·37)	0·4407
Cough		113/346 (34%)	108/326 (33%)	105/329 (32%)	0·99 (0·62 to 1·60)	0·9827	0·88 (0·55 to 1·41)	0·5865	0·88 (0·55 to 1·42)	0·6058
Swelling of legs or ankles		95/349 (27%)	89/324 (27%)	80/326 (25%)	1·22 (0·66 to 2·26)	0·5279	0·75 (0·40 to 1·41)	0·3763	0·62 (0·33 to 1·16)	0·1338
Sore eyes		80/348 (23%)	86/324 (27%)	71/325 (22%)	1·07 (0·64 to 1·79)	0·7861	0·83 (0·49 to 1·41)	0·4965	0·78 (0·46 to 1·32)	0·3462
Dry mouth		66/348 (19%)	87/324 (27%)	71/326 (22%)	2·64 (1·37 to 5·07)	0·0036	1·23 (0·63 to 2·39)	0·5427	0·47 (0·24 to 0·89)	0·0208
Pins and needles		69/346 (20%)	76/325 (23%)	76/328 (23%)	1·02 (0·53 to 1·98)	0·9542	1·00 (0·52 to 1·94)	0·9941	0·98 (0·51 to 1·90)	0·9599
Loss of strength		75/347 (22%)	66/325 (20%)	77/328 (23%)	0·70 (0·38 to 1·30)	0·2615	0·95 (0·52 to 1·74)	0·8678	1·35 (0·72 to 2·53)	0·3426
Hypertension-specific symptoms not in top 10										
	Feeling flushed	73/346 (21%)	66/324 (20%)	76/328 (23%)	0·76 (0·41 to 1·41)	0·3776	0·96 (0·52 to 1·76)	0·8854	1·26 (0·68 to 2·34)	0·4572
	Dizziness	61/348 (18%)	50/324 (15%)	72/326 (22%)	0·77 (0·38 to 1·54)	0·4546	1·40 (0·72 to 2·74)	0·3212	1·83 (0·92 to 3·65)	0·0869
	Impotence	37/310 (12%)	43/288 (15%)	43/274 (16%)	1·85 (0·42 to 5·22)	0·2422	1·01 (0·34 to 3·05)	0·9789	0·5475 (0·18 to 1·57)	0·2633

Data are n/N of total responses (%), unless otherwise stated.

* Total number attending 12-month follow-up.

† Significant at p<0·017.

There was no evidence of a non-pharmacological effect of self-monitoring or telemonitoring in terms of diet, exercise, smoking, or alcohol consumption (appendix p 7). Similarly, weight was not significantly different between baseline and follow-up in either intervention group compared with the usual care group. Quality of life, as measured by the EQ-5D-5L,^26^ was not significantly different between each group at 12 months follow-up (appendix p 7).

The number of primary care consultations during the year of the study were similar between all three groups with slightly higher consultation rates in the first 6 months of the trial (appendix p 8). More clinic blood pressure readings were taken in the usual care group compared with the self-monitoring or telemonitoring groups.

## Discussion

This trial has shown that the use of self-monitoring of blood pressure in primary care to titrate antihypertensive therapy for the management of individuals with poorly controlled hypertension in primary care results in lower systolic blood pressure without increasing GP workload. After 1 year, patients whose medication was adjusted using self-monitoring, with or without telemonitoring, had significantly lower systolic blood pressure than those receiving treatment adjusted using clinic blood pressure. There was no evidence of a differential effect in the subgroups examined, including importantly no difference by age.

This trial was not powered to detect cardiovascular outcomes, but the differences between the interventions and control in systolic blood pressure would be expected to result in around a 20% reduction in stroke risk and 10% reduction in coronary heart disease risk.^10^, ^27^ Although not significantly different from each other at 12 months, blood pressure in the group using telemonitoring for medication titration became lower more quickly (at 6 months) than those self-monitoring alone, an effect which is likely to further reduce cardiovascular events and might improve longer term control.^28^, ^29^

No evidence was found regarding increased adherence as measured by the MARS scale, but this might have been due to a ceiling effect as scores were above 95% of the maximum in each group.^23^ A recent systematic review found that self-monitoring increased adherence to antihypertensives, at least when measured by more objective means that were beyond the resources available in this study.^30^, ^31^ The results from prescribing records (table 3) suggest that the mechanism of action was differential intensification of antihypertensive medication in response to self-monitored blood pressure, although this was modest. No evidence of other non-pharmacological effects from self-monitoring were found, at least in terms of self-reported diet, exercise, or alcohol intake. Importantly, there were no differences in side-effects or quality of life despite greater medication use in the intervention groups.

This study was undertaken in a large population drawn from primary care across the UK. Patients were recruited from a wide variety of practices, had a mean blood pressure of 153/86 mm Hg, and 60% had another ongoing medical condition. However, just over half of those assessed for the trial were not eligible, largely because of having controlled blood pressure. The average age of participants was 67 years and approximately equal numbers of men and women took part, but most were white British ethnicity, which might limit generalisability. The commonest reason for exclusion from the trial was controlled blood pressure and the vast majority—around 90%—of those with uncontrolled blood pressure were randomised suggesting generalisability in this group.

Overall, the trial followed-up 85% of those randomised but there were, albeit non-significantly, fewer seen at final follow-up in both intervention groups compared with control (83% *vs* 88%), perhaps reflecting increased withdrawal due to the self-monitoring intervention. Despite this, recruitment was slightly higher than planned and the numbers in all three study groups comfortably exceeded the 315 participants per group required in the prespecified power calculation.^16^ Furthermore, sensitivity analyses using imputation gave similar results to those in the primary complete case analysis, suggesting that differential follow-up was unlikely to have biased the headline results.

Ambulatory monitoring might have been a superior outcome measurement in terms of better correlation with cardiovascular outcome and reduced white coat effect, but would have raised substantial barriers in terms of logistical and financial challenges.^32^ The use of multiple blood pressure measurements in the sensitivity analysis formed a proxy for ambulatory monitoring and, as with other work from this group, no evidence was found of habituation to repeated blood pressure measurement.^8^, ^9^ Use of clinic measurements at baseline will have potentially excluded those with masked uncontrolled hypertension.^33^ There was no evidence of increased anxiety from self-monitoring,^21^ something which is often mentioned by health-care professionals but which has not been detected in trials by this group to date.^34^

Clinician consulting rates for hypertension over the year were similar in all three groups (mean consultations [95% CI]: usual care, 2·1 [1·9 to 2·3]; self-monitoring, 1·8 [1·6 to 2·1]; and telemonitoring, 2·2 [2·0 to 2·5]), but given that clinicians were asked to review intervention groups' blood pressure on a monthly basis, might have failed to capture additional workload associated with self-monitoring.

We have recently published an individual patient data meta-analysis of self-monitoring in the control of blood pressure, and to our knowledge, this is the first study to directly compare GPs titrating antihypertensives using self-monitoring with or without telemonitoring, with usual care in hypertensive patients, using appropriately lower home targets and followed-up for 12 months.^10^ Previous studies by Staessen and colleagues^12^ and Verberk and colleagues^13^ found worse clinic blood pressure control and less treatment when using identical treatment targets for both home and clinic blood pressures. The current study used nationally and internationally recommended self-monitoring targets, 5 mm Hg lower for both systolic and diastolic values, and found better control and more utilisation of antihypertensive medication.

This work extends the evidence from McKinstry and colleagues^15^ using a telemonitoring based service design over 6 months by following up for a full year. Differences in blood pressure recorded at 6 months were amplified by a year suggesting that the intervention increased in efficacy in the second 6 months. The individual patient data meta-analysis found that the intensity of self-monitoring co-interventions was related to the effect on blood pressure and the quicker reduction in blood pressure with telemonitoring guided titration is in line with this.^10^ An economic analysis and qualitative study will follow this work and will be important in understanding the place of telemonitoring over and above self-monitoring in the management of hypertension.

No trial of self-monitoring to date has been powered for clinical events but blood pressure reduction is extremely well correlated with reduced morbidity and mortality.^27^ Furthermore, self-monitored blood pressure is better correlated with cardiovascular outcomes than clinic blood pressure and so treatment guided by self-monitoring might be expected to have at least the effect seen in clinic blood pressure trials, with the advantage of not unnecessarily treating white coat hypertension, whereas conversely addressing masked uncontrolled hypertension.^33^, ^35^ Arrangements have been made to follow the patients in this trial in the longer term for mortality and, in combination with other trials, it might be possible to use these data to make judgments on the effect of self-monitoring interventions on hard outcomes. In the meantime, a very large multicentre, perhaps international, trial of self-monitoring would be needed to detect differences in such outcomes.

Telemonitoring reminded participants if they had not monitored sufficiently and prompted contact with their GP for out-of-range readings. Health-care professionals were provided with mean blood pressure (rather than having to calculate by hand) along with a graphical presentation of trends in blood pressure. This provision might be expected to be advantageous in situations where doctors feel overwhelmed by the quantities of data they are required to deal with on a daily basis. Whether such advantages over self-monitoring alone are clinically worthwhile will be informed by the economic analysis. The text-based telemonitoring system was simple and could be implemented in other health systems as it was not dependent on a particular mobile phone provider or clinical system. The feedback system could be adapted into a smartphone application and could be incorporated into clinical patient record systems. Furthermore, it might be expected to offer particular advantages to the working age population where telemonitoring might facilitate management and remote access to a physician.

By contrast, self-monitoring alone is simple, can be accomplished with a cheap validated monitor, and produced similar results to telemonitoring, albeit with less effect at 6 months. However, although the evidence supports use, the logistical problems of sending back results by post, and manual data processing and recording of results, suggests that comprehensive implementation outside a research study might be challenging.

Blood pressure self-monitoring is now commonplace in the UK and elsewhere and practised by at least 30% of people with hypertension.^36^, ^37^ A recent survey of primary-care physicians suggested that many are already taking advantage of self-monitored blood pressure results in a proportion of their patients and the current work provides evidence to underpin and expand this.^11^ Participants in both the self-monitoring and telemonitoring groups reported equivalent quality of life compared with usual care and no increase in consultation rates. The success in controlling blood pressure from the use of self-monitoring to titrate antihypertensives, and a lack of adverse events, suggests that the technology is now ripe for wider implementation in daily practice and in national and international guidance. Other countries might need to consider changes in reimbursement systems that currently favour (and reward) face-to-face contact.^38^ Guidelines have recently recommended lower targets for most people with hypertension and self-monitoring provides one means of achieving better control, although it should be emphasised that the final mean blood pressures in both intervention groups suggest that a proportion of participants will still have been above even a 140/90 mm Hg target.^39^

Titration of antihypertensive medication in primary care using self-monitoring, with or without telemonitoring, results in significantly lower blood pressure after 1 year and using telemonitoring leads to lower blood pressure after 6 months. Self-monitoring can be recommended for the ongoing management of hypertension in primary care in all patients who wish to use it, and will require provision of validated blood pressure monitors for home use, ideally with integrated telemonitoring systems. GPs should incorporate self-monitored readings into their titration of blood pressure medications.
