# Supplementary material for: Efficacy of self-monitored blood pressure, with or without telemonitoring, for titration of antihypertensive medication (TASMINH4): an unmasked randomised controlled trial
Source: Lancet. 2018 Mar 10;391(10124):949–59. doi: 10.1016/S0140-6736(18)30309-X (PMC5854463; doi:10.1016/S0140-6736(18)30309-X)
Supplement: Supplementary appendix [file mmc1.pdf]

THE LANCET

Supplementary appendix

This appendix formed part of the original submission and has been peer reviewed. We post it as supplied by the authors.

Supplement to: McManus RJ, Mant J, Franssen M, et al. Efficacy of self-monitored blood pressure, with or without telemonitoring, for titration of antihypertensive medication (TASMINH4): an unmasked randomised controlled trial. *Lancet* 2018; published online Feb 27. http://dx.doi.org/10.1016/S0140-6736(18)30309-X.

Appendix

The TASMINH4 Investigators 2

Primary Care Clinical Trials Unit Oxford: 2

Research Nurses/Facilitators: 2

Recruiting Practices and Lead GPs: 3

Additional Tables 5

Table A1 Detailed Reasons for Exclusion (See CONSORT Diagram) 5

Table A2 Adjusted Results For The Systolic And Diastolic Blood Pressure Differences At 12 Months Under Different Sensitivity Analyses 6

Table A3 Mean Differences At 12 Months for Quality of Life, Anxiety, Weight Diet, Alcohol Intake, Smoking and Exercise Scores 7

Table A4 Practice Utilisation During the Trial 8

Algorithm details 9

Figure A1: Colour coding chart (non-diabetic patients under 80 years old) 10

Statistical Analysis Plan 11

1 Introduction 12

2 Data Monitoring Committee And Interim Analyses 19

3 Primary Analysis 19

4 Secondary Analysis 21

5 Subgroup Analyses 24

6 Sensitivity analysis 24

7 Safety Analysis 25

8 Changes to protocol or previous versions of SAP 25

9 Appendices 26

The TASMINH4 Investigators

Primary Care Clinical Trials Unit Oxford:

Brendan Bradley (Data Management); Chris Lovekin (Data Management); David Judge (Programming); Luis Castello (Programming); Maureen Dawson (Administrative Support); Rebecca Brice (Administrative Support); Bethany Dunbabin (Administrative Support).

Research Nurses/Facilitators:

Thames Valley

Sophie Maslen; Heather Rutter; Mary Norris; Lauren French; Michael Loynd; Pippa Whitbread; Luisa Saldana Ortaga; Irene Noel; Karen Madronal.

Birmingham and the Black Country

Julie Timmins; Peter Bradburn; Lucy Hughes; Beth Hinks; Sheila Bailey; Sue Read; Andrea Weston; Somi Spannuth; Sue Maiden; Makiko Chermahini; Ann McDonald.

Eastern

Shelina Rajan; Sue Allen; Brenda deBoys; Kim Fell; Jenny Johnson; Helen Jung; Rachel Lister; Ruth Osborne; Amy Secker.

West Midlands South

Irene Qasim; Kirsty William; Abi Harris; Susan Zhao; Elaine Butcher; Pauline Darbyshire; Sarah Joshi; Jon Davies; Claire Talbot; Eleanor Hoverd; Linda Field; Tracey Adcock.

Kent Surry and Sussex

Julia Rooney; Nina Cooter; Aaron Butler; Naomi Allen.

West of England
Maria Abdul-Wahab; Kathryn McNicholas ; Lara Peniket.

North West
Kate Dodd; Julie Mugurza.

Recruiting Practices and Lead GPs:

27 Beaumont Street, Dr Richard Baskerville; Aksyr, Dr Rakshan Syed; Arden, Dr Clare Bailey; Arun, Dr Jill Adams; Ashfields Primary Care Centre, Dr Carolyn Paul; Barn Close, Dr Neil Townsend; Bay Medical Group, Morecambe Bay, Dr Alison Macleod; Beccles Medical Centre, Dr Charlotte Hawkins; Bennfield Surgery, Dr Suparna Behura; Berinsfield Health Centre, Dr Jonathan Crawshaw; Bicester Health Centre, Dr Robin Fox; Bournville, Dr Waleed Doski; Bridge Road Surgery, Dr Martin Aylward; Broadshires Health Centre, Dr Christine A'Court; Castle Medical Centre, Dr David Rapley; Castle Partnership, Dr Jo Walsh; Chancery Lane, Dr Paul Batra; Charlotte Keel Medical Practice, Ana Seoane; Cherry Hinton Surgery, Dr Sluti Mukherjee; Churchdown Surgery, Dr Jonathan Dixon; Claughton Medical Centre, Dr Peter Arthur; Cleveleys Group Practice, Karen Sutcliffe; Colchester Medical Practice, Dr C Paschallides; Corbett, Dr Richard Woof; Cornerstone, Dr Peter Winfrey; Cranleigh, Dr Matthew Clark; Cubbington Road, Dr Roya Kamali; Cuckoo Lane, Dr Paul Thomas; Didcot, Dr David Ebbs; Doddington, Dr Liz Mather; Dorking, Dr Andre Beattie; Dovecote, Dr Karim Ladha; Dr Jeffries and Partners, Dr Larisa Smondulak**;** Drs Pal and Jemahl, Dr Surinder Jemahl; Dr Singh and Partners, Dr Peter Hickson; East Norfolk, Dr Liam Stevens; Elm Tree, Dr Tony Crockett; Eve Hill, David Shukla; Eynsham, Dr Ian Binnian; Furnace Green, Dr Paul Vinson; Gladstone, Dr Nigel DeKare-Silver; Grange Hill, Dr Ramila Patel; Greenbrook Isleworth, Dr Ivor Singh ; Greenridge, Dr Louise Lumley; Grove Park Terrace, Dr Glennis Williams; Grove Road, Dr Mark Webb; Holbrooks, Dr Jack Bambrough; Ivers Practice, Dr Neetul Shah; Jubilee-Broad Street, Dr Hergeven Dosanjh; Kingsfield, Dr Frank Spannuth; The Kiltearn Medical Centre, Dr Carolyn Paul; Langley Corner, Dr Jude Ganesegaram; Laurie Pike**,** Dr Vijaysundari Maheswaran**;** Leighton Road, Dr Farah Paruk; Lensfield Road, Dr Stephen Ford; Limes, Dr Vineeta Verma; Magdalen, Dr Kate Milne; Manor Court, Dr Farhana Lockhat; Marlow, Dr Jennifer Ferguson; Medwyn, Dr Anne-Marie Quirk; Mid Sussex, Dr Hugo Wilson; Morland House, Dr David Copping; Moseley Avenue, Dr Bajallan; North Hyde Road Surgery, Dr Simria Tanvir; Norvic, Dr Faheem Khan; Nuffield Road, Dr Tom Alderson; Oakenhurst Medical Practice, Dr Amar Ali; Paston, Dr Richard Young; Pendle View Medical Centre, Dr Umesh Chauhan; Peninsula Practice, Dr Lindsey Crockett; Poplar, Dr Louise McGovern; Prospect, Dr Claire Cubitt; Queen Square Medical Practice, Lancaster, Dr Simon Weatherill; Regis, Dr Abdul Tabassum; Ridgacre, Dr Philip Saunders; Riverbrook, Dr Naresh Chauhan ; Riversley Road, Dr Samantha Johnson; Rosedale Surgery, Dr Jo Walsh; Rotton Park, Dr Inderjit Marok; Sea Road, Dr Sharma; Sedbergh Medical Practice, Dr William Lumb; Shelford, Dr Tweedale; Sheringham, Dr Ian Smith; Sherwood, Dr Lawrence Miller; Shifa Surgery, Dr Tanveer Ahmed; Spinney Partnership, Dr Mark Sanderson; Spring Gardens, Dr Claire Jones; St Catherine's Surgery, Dr

Peter Stokell; St James, Dr Matthew J Edwards; St John, Dr Andrew Askey; St Martin's Gate, Dr Jason Spencer; Station House Surgery, Kendal, Dr Kathryn Morgan; Summertown, Dr Kyle Knox; The Grange, Dr Robert Baker; The Marches, Dr Crispin Fisher; The Royle Practice, Blackburn, Dr Rachel Halstead; Thistlemoor, Dr Neil Modha; Trinity Court, Dr David Buckley; Victoria Park, Dr Peter Stokell; Wallace House, Dr John Gerald McCabe; Wand, Dr Jennifer Taylor; Warley, Dr Helen Nutbeam; Wellside, Dr Richard Smith; West Bar, Dr Christopher MacGregor; West Walk, Dr Sam Davies; Westside, Dr Mark Lindsey.; White Horse, Dr Simon Cartwright; White House, Dr Jonathan Whittle; Windermere, Dr Julie Colclough, Dr Alison Crumbie; Windrush, Dr Nicholas Thomas; Winyates, Dr Vattakkatt Premchand; Wish Park, Dr Rafia Hamid; Wokingham, Dr Zishan Ali; Woodgate Valley, Dr John Ward; Wymondham, Dr Philip Pinney, Dr Stephen Thurston; Yardley Wood, Dr Tina Banerjee.

Additional Tables

Table A1 Detailed Reasons for Exclusion (See CONSORT Diagram)

Reasons for exclusion	Not randomised
Blood pressure < 140/90mmHg	1048
>3 Hypertensive medications	7
Orthostatic hypertension	86
Atrial fibrillation	9
Unwilling to self-monitor	5
Blood pressure managed outside of GP practice	8
Unable to provide consent	5
Dementia	3
Female pregnant or lactating	0
Chronic kidney disease at stage 4 or 5 or Chronic kidney disease with proteinuria	7
Other disease	1
Has not had a stable dose of hypertensive medication	22
Other	22
Note: Reasons not mutually exclusive. 1036 participants had one identifiable reason for exclusion, 158 participants had two reasons, and seven participants had three reasons for exclusion. Most reasons listed under “other” related to inability to obtain a blood pressure reading at the appointment.	

Table A2 Adjusted Results For The Systolic And Diastolic Blood Pressure Differences At 12 Months Under Different Sensitivity Analyses

	Self-Monitoring vs usual care		Telemonitoring vs usual care		Telemonitoring vs Self-Monitoring	
	**Adjusted Mean Difference Between Randomised Groups (95% CI) (units = mmHg)**	**p-value[+]**	**Adjusted Mean Difference Between Randomised Groups (95% CI) (units = mmHg)**	**p-value[+]**	**Adjusted Mean Difference Between Randomised Groups (95% CI) (units = mmHg)**	**p-value[+]**
**Systolic blood pressure at 12 months (mean of 2-6th readings) controlling for habituation***	-3.87 (-6.05; -1.69)	0.0005	-4.96 (-7.13; -2.78)	< 0.0001	-1.09 (-3.30; 1.12)	0.3357
**Systolic blood pressure at 12 months (mean of 2-3rd readings) including participants missing one of 2nd or 3rd measurement***	-3.49 (-5.81; -1.17)	0.0032	-4.64 (-6.96; -2.33)	0.0001	-1.16 (-3.50; 1.19)	0.3345
**Systolic blood pressure at 12 months (mean of 2-3rd readings) adjusting for factors associated with missingness[#]**	-3.62 (-5.94; -1.30)	0.0022	-4.67 (-6.99; -2.35)	0.0001	-1.05 (-3.41; 1.31)	0.3825
**Systolic blood pressure at 12 months (mean of 2-3rd readings) imputing missing blood pressures with nearest neighbours**	-3.54 (-5.86; -1.23)	0.0027	-4.77 (-7.08; -2.45)	0.0001	-1.23 (-3.57; 1.12)	0.3062
**Systolic blood pressure at 12 months (mean of 2-3rd readings) imputing missing blood pressures via multiple imputation**	-3.50 (-5.80; -1.18)	0.0030	-4.69 (-6.99; -2.38)	< 0.0001	-1.19 (-3.53; 1.15)	0.3180
**Systolic blood pressure at 12 months (mean of 2-3rd readings) excluding likely erroneous blood pressure readings**	-3.48 (-5.80; -1.17)	0.0032	-4.59 (-6.90; -2.27)	0.0001	-1.10 (-3.45; 1.24)	0.3567
**Diastolic blood pressure at 12 months (mean of 2-6th readings) controlling for habituation***	-1.38 (-2.52; -0.24)	0.0178	-1.32 (-2.46; -0.18)	0.0233	0.06 (-1.10; 1.22)	0.9175

*Linear mixed effect model of blood pressure at 6 and 12 months modelled against randomised group, time of visit and its interaction with randomised group, baseline blood pressure, gender, history of CVD, target blood pressure as fixed effects and practice as a random effect
#Linear mixed effect model of systolic blood pressure at 6 and 12 months modelled against randomised group, time of visit, baseline systolic blood pressure, gender, history of CVD, target blood pressure, mobility problems and smoking as fitted effects and practice as a random effect

*Level of significance = 0.017 to account for multiple comparisons between intervention groups

Table A3 Mean Differences At 12 Months for Quality of Life, Anxiety, Weight Diet, Alcohol Intake, Smoking and Exercise Scores.

	Self-Monitoring vs usual care		Telemonitoring vs usual care		Telemonitoring vs Self-Monitoring	
	**Adjusted Treatment Difference (95% CI)**	**p-value+**	**Adjusted Treatment Difference (95% CI)**	**p-value+**	**Adjusted Treatment Difference (95% CI)**	**p-value+**
**Quality of life*# (EQ-5D-5L)**	-0.01 (-0.04; 0.02)	0.4862	-0.03 (-0.06; -0.001)	0.0384	-0.02 (-0.06; 0.01)	0.2238
**STAI-6 +**	0.07 (-2.60; 2.75)	0.9563	0.37 (-2.32; 3.05)	0.7884	0.29 (-2.43; 3.01)	0.8329
**Weight***	0.49 (-0.05; 1.04)	0.0761	0.10 (-0.44; 0.64)	0.7182	-0.39 (-0.95; 0.16)	0.1633
**Diet score* 12 months**	1.20 (0.06; 2.34)	0.0394	0.93 (-0.20; 2.07)	0.1068	-0.27 (-1.43; 0.90)	0.6538
**Exercise score* 12 months**	5.57 (-1.07; 12.2)	0.1002	1.30 (-5.4; 7.98)	0.7019	-4.26 (-11.00; 2.47)	0.2150
**Alcohol score* 12 months**	-0.02 (-0.25; 0.20)	0.8328	-0.02 (-0.24; 0.20)	0.8550	0.00 (-0.22; 0.23)	0.9769
**Smoking$**	0.92 (0.70; 1.20)	0.5490	0.87 (0.67; 1.12)	0.2972	0.9456 (0.70; 1.28)	0.7158

*Linear mixed effect model of the outcome score at 12 months modelled against randomised group, time of visit and its interaction with randomised group, baseline outcome score, baseline systolic blood pressure, gender, history of CVD, target blood pressure as fixed effects and practice as a random effect

$ Generalised linear model with a log-link function (log-binomial model) of the outcome at 12 months modelled against randomised group, baseline outcome score

#Data were skewed bootstrapped standard errors and confidence intervals were derived

+Level of significance = 0.017 to account for multiple comparisons between intervention groups

+ 6 point State Anxiety Questionnaire

Table A4 Practice Utilisation During the Trial

	Usual Care	Self-Monitoring	Telemonitoring
**Mean number of hypertension consultations (95% CI) between baseline and 6 months follow-up**	1.3 (1.2. 1.0)	1.1 (0.9, 1.2)	1.3 (1.2, 1.5)
**Mean number of hypertension consultations (95% CI) between 6 and 12 months follow-up**	0.8 (0.7, 1.0)	0.8 (0.6, 0.9)	0.9 (0.7. 1.0)
**Mean number of hypertension consultations (95% CI) between baseline and 12 months follow-up**	2.1 (1.9, 2.3)	1.8 (1.6, 2.1)	2.2 (2.0, 2.5)
**Average number of BP readings taken at their GP surgery between baseline and 6 months follow-up**			
Mean (sd)	1.1 (1.43)	0.6 (1.1)	0.9 (1.4)
Median (IQR)	1 (0; 2)	0 (0; 1)	0 (0; 1)
**Average number of BP readings taken at their GP surgery between 6 months and 12 months follow-up**			
Mean (sd)	0.8 (1.4)	0.5 (1.3)	0.6 (1.3)
Median (IQR)	1 (0; 2)	0 (0; 1)	0 (0; 1)
**Average number of BP readings taken at their GP surgery between baseline and 12 months follow-up**			
**Mean (sd)**	1.9 (2.2)	1.1 (1.8)	1.5 (2.1)
**Median (IQR)**	1 (0; 3)	0 (0; 1)	1 (0; 2)
**N (%) having blood pressure readings recorded in GP / PN surgery**			
6 Months	228 (58.0%)	127 (32.5%)	166 (42.7%)
12 Months	168 (42.8%)	106 (27.1%)	124 (31.9%)
**Mean (sd) systolic blood pressure reading in GP / PN surgery**			
6 Months	144.8 (13.7)	143.8 (13.3)	143.3 (17.0)
12 Months	145.9 (16.0)	139.5 (18.2)	141.4 (18.0)
**Mean (sd) diastolic blood pressure reading in GP / PN surgery**			
6 Months	80.5 (9.2)	83.7 (8.5)	83.42 (9.4)
12 Months	81.6 (9.2)	79.6 (8.6)	80.86 (9.2)
[1]Target BP for home readings (135/85 mmHg for standard, 145/85 mmHg for older, and 135/75 mmHg for diabetes)			
[2]Target BP for GP readings (140/90 mmHg for standard, 150/90 mmHg for older, and 140/80mmHg for diabetes)			

Algorithm details

Participant training included instructions as to what to do in the presence of a high or low reading, using a guideline that contains simple colour-coded instructions (see Figure A1). These are reproduced below and were based on those developed for the TASMINH2 and TASMIN-SR trials (McManus et al Lancet 2010 and JAMA 2014 respectively). Instructions asked the patient to contact their practice for very high or very low readings that persisted when a third reading was taken five minutes after the second reading. Thresholds for raised readings varied depending on the participant characteristics (non-diabetic <80 years, diabetic, 80+ years) but high and low safety thresholds were identical for all.

Participants randomised to the telemonitoring group sent their readings to a secure centralised database using a free SMS text message with web-based data entry back up. The telemonitoring system effectively operationalised the colour chart (Figure A1), but key differences from the self-monitoring alone group were that:

a) They received a reminder the day before their week of measurements, and one additional reminder in the week if no measurements are received by the system. If at the end of the week, insufficient readings to calculate a mean had been received (defined as 12 readings in the week, ignoring the first day), a further reminder to complete outstanding readings was sent.
b) Each time a reading was sent successfully, the system acknowledged it.
c) At the end of each monitoring week, mean BP was calculated automatically from available readings (provided at least 12 readings had been received after the first day) and participants received an SMS message either confirming that their blood pressure was controlled and to keep on monitoring or requesting that they made an appointment with their GP or nurse to review their medication.
d) High or low readings (red (>170/105mmHg) or blue zones (<100mmHg systolic as per above colour chart), triggered text alerts to the patient to recheck the reading and then contact their surgery for a BP check if it remained high.

In both self-monitoring groups, the GP/ nurse was asked to review the readings on a monthly basis via either paper- based forms submitted by patients or through the web-based interface. They were asked to determine whether a change in medication was required and to contact the patient if a medication change was required. The web-based interface highlighted patients with either safety triggers or whose blood pressure was above target. GPs could use the web interface to respond to patients via SMS.

NUFFIELD DEPARTMENT OF PRIMARY CARE HEALTH SCIENCES — UNIVERSITY OF BIRMINGHAM — TASMINH4

UNDERSTANDING YOUR MEASUREMENTS

For non-diabetic patients under 80 years

For RED or BLUE readings you will need to repeat them initially and if they remain too high or low you will be advised to seek medical advice.

In each case, the top reading is the SYStolic and bottom reading DIAstolic.

Colour	Level	Blood Pressure	Action
RED	HIGH	SYS 171 or more<br>OR<br>DIA 106 or more	**Your BP is too high.**<br>Make an appointment within 48 hours to see your GP or nurse.
AMBER	RAISED your GP/nurse may contact you to alter your medication	SYS 136-170<br>OR<br>DIA 86-105	**Your BP is raised.**<br>If you have persistent AMBER readings then you will receive contact from your GP/Practice nurse as you may need your medication altered.
GREEN	NORMAL	SYS 100-135<br>AND<br>DIA 85 or less	**Your BP is normal.**<br>This is fine provided that you have no side effects.
BLUE	LOW	SYS 99 or less	**Your BP is too low.**<br>Make an appointment within 48 hours to see your GP or nurse.

Figure A1: Colour coding chart (non-diabetic patients under 80 years old)

Statistical Analysis Plan

Title: Telemonitoring and/or self-monitoring of blood pressure in hypertension: A randomised controlled trial in primary care.
Short title: TASMINH4
Ethics Ref: 14/SC/0218
Version Number and date: Version 1.0 14 March 2017

	NAME	TITLE
Written by:	Jill Mollison	Senior Trial Statistician
Reviewed by:	Ly-Mee Yu	Lead Trial statistician
Approved by:	Richard McManus	Chief Investigator

<u>Version History</u>

Version:	Version Date:	Changes:

1 Introduction

1.1 Preface

Chief Investigator: Professor Richard McManus
This SAP supports ‘TASMINH4 protocol V2.1 19/05/2016’.

1.2 Purpose and scope of the plan

This document details the proposed analysis of the main paper(s) reporting results from the TASMINH4 NIHR funded randomised controlled trial to evaluate the management of hypertension in primary care using self monitored blood pressure, with or without telemonitoring compared with standard care. The results reported in these papers should follow the strategy set out here. Subsequent analyses of a more exploratory nature will not be bound by this strategy, though they are expected to follow the broad principles set out here. The principles are not intended to curtail exploratory analysis (for example, to decide cut-points for categorisation of continuous variables), nor to prohibit accepted practices (for example, data transformation prior to analysis), but they are intended to establish the rules that will be followed, as closely as possible, when analysing and reporting the trial.
The analysis strategy will be available on request when the principal papers are submitted for publication in a journal. Suggestions for subsequent analyses by journal editors or referees, will be considered carefully, and carried out as far as possible in line with the principles of this analysis strategy; if reported, the source of the suggestion will be acknowledged.
Any deviations from the statistical analysis plan will be described and justified in the final report of the trial. The analysis should be carried out by an identified, appropriately qualified and experienced statistician, who should ensure the integrity of the data during their processing. Examples of such procedures include quality control and evaluation procedures.
The details regarding analysis of the economic sub study will be detailed in a separate analysis plan.

1.3 Trial overview

Blood pressure is a key risk factor for cardiovascular disease. BP control within the population is sub optimal and factors responsible for this include those due to patients, physicians and the health system. Self-monitoring of hypertension may be a solution.

1.4 Objectives

Primary objective

1. To evaluate the management of hypertension in primary care using self-monitored blood pressure, with or without telemonitoring compared to usual care.

Secondary objectives

1. Is the effect seen for conventional systolic blood pressure (SBP) consistent for diastolic and does it remain when controlling for habituation to repeated measurement?

Tertiary objectives

1. What is the effect of self-monitoring on adherence, side effects, quality of life, adverse events, lifestyle behaviour and costs?

2. Is it possible to use routine GP clinical systems to collect sufficiently robust data for a subsequent trial powered on cardiovascular outcomes?

1.5 Trial design

TASMINH4 is a pragmatic un-blinded individual patient randomised controlled trial. At least 1110 patients with hypertension recruited from primary care will be randomised into one of the three groups: self-monitoring alone, self-monitoring with telemonitoring, and usual care (control) (1:1:1).For all analyses, the three randomised groups will be compared in the following way:

(i) Self-monitoring alone versus usual care
(ii) Self-monitoring with telemonitoring versus usual care
(iii) Self-monitoring alone versus self-monitoring with telemonitoring

The **study population** will comprise people with poorly controlled hypertension managed in primary care. Eligibility criteria will be aged over 35 years, on the hypertension register, not already taking more than three anti-hypertensive agents, BP above 140/90 mmHg at the baseline clinic, and on a stable dose of current antihypertensive medication for at least four weeks prior to trial entry. Exclusion criteria will be orthostatic hypertension (20 mmHg or more systolic drop after standing for one minute, in order to avoid adverse events), BP not managed by their GP (limited possibility of antihypertensive titration), diagnosed atrial fibrillation (automated monitors not validated), unwilling to self-monitor, dementia or score over 10 on the short orientation memory concentration test (inability to undertake self-monitoring), female participant who is pregnant, lactating or planning pregnancy during the trial (management of essential hypertension in pregnancy is different), the partner or spouse of an individual already randomised in the trial (to avoid clustering within families), Chronic Kidney Disease (CKD) grade four or worse, any grade of CKD with proteinuria (both may have different BP targets), participants who have participated in another research trial involving antihypertensive medication in the past four weeks.

See Appendix I for a time schedule of trial procedures

1.6 Outcomes measures

Outcome measures are assessed at baseline and follow up (6 and 12 months) by the research team in the patients' own practice. Data will be collected onto paper questionnaires.
See Appendix II for a table of outcomes assessment schedule.

1.6.1 Primary outcome

The primary outcome is systolic blood pressure at 12 months.

Six measurements of BP are taken at baseline and each follow up visit. The mean of the 2nd and 3rd BP readings (conventional BP) will be used in the primary outcome assessment.

1.6.2 SECONDARY/TERTIARY OUTCOMES

Secondary outcomes
To examine whether the effect seen for conventional systolic blood pressure is consistent for diastolic blood pressure (ii) and does it remain when controlling for habituation to repeated measurements (iii).

(i) Systolic BP at six months (mean of 2nd/3rd measurements – *SYSBP23*).

(ii) Diastolic blood pressure at six and 12 months (mean of 2nd and 3rd measurements – *DIABP23*).

(iii) The analyses (Systolic and diastolic BP at 6 and 12 months) will be repeated controlling for habituation. The mean of the 2-6th measurements (*SYSBPMN, DIABPMN*) will be used. If ONLY one reading is missing, due to machine error, the mean of four available 2-6th measurements will be used in the analysis.

Tertiary outcomes
To examine the effect of self monitoring on adherence, side effects, quality of life, adverse events, lifestyle

(iv) Adverse Events

a. **Clinical Events**: Admission to hospital, cardiovascular events and deaths will be recorded as part of the safety monitoring and for the economic analysis. Cardiovascular events and deaths. The patient's clinical records will be reviewed at 6 and 12 months follow up and any cardiovascular events will be collected. Patients will also be flagged for mortality at the NHS central register. Following the final follow up, data will be extracted using the clinical records / Clinical Practice Research Datalink (CPRD) on death, risk factors and cardiovascular disease in order that death risk factor recording and cardiovascular disease coding can be validated. In addition up to 10 years of ONS data will be obtained in the future to assess the long-term mortality outcomes for these participants and the analysis of this is not covered in this statistical analysis plan.

b. **Anxiety** is measured using the short form of the State Trait Anxiety Inventory (**STAI**) at 6 months and 12 months follow up. The short form STAI includes 6 statements with responses from 'not at all' to 'very much'. Responses are scored as 'not at all' = 1 to 'very much' =4. Total score ranges from 6 to 24. These are scaled to be out of 100 to allow comparison to the full version of STAI for which population norms are published.

c. **Side effects / Symptoms**: Section 6 in the 6 and 12 month follow up symptom questionnaires assesses whether the patient has recently experienced the 24 symptoms listed (Yes/No).

(v) Medication Outcomes

a. **Current medications**

Data on current medications will be converted into defined daily doses. The DDD is the assumed average maintenance dose per day for a drug used for its main indication in adults and is defined by the WHO (http://www.whocc.no/ddd/definition_and_general_considera/).
The change is DDD will be computed between baseline and 6 months and baseline and 12 months. Data will be presented separately for intervention and control.

b. **Patient adherence** to prescribed medication will be assessed using the Medication Adherence Rating Scale (**MARS**) at 12 months. The MARS is a 5-item self-report scale, the items are rated on a 5-point Likert scale, ranging from 1='always' to 5 = 'never' (range 5 to 25). Higher scores indicate lower levels of adherent behaviour. MARS will produce highly skewed data, so that the baseline measures will be presented using median and quartiles.

c. **Beliefs about medicine (BMQ)**The beliefs about medicines questionnaire (BMQ-Specific) includes 10 statements regarding their views about their blood pressure medication assessed using a 5 point Likert scale (strongly agree =5 to strongly disagree=1). There are two subscales – necessity (Questions a,c,d,g,j) and concerns (questions b,e,f,h,i) each has a range of 5 to 25 calculated by summing the scores. Studies using the BMQ-specific sub-scales have shown that respondents with stronger beliefs about the necessity of their medication and fewer concerns about their medication (as measured by the BMQ) are more likely to use their medicines as recommended by the prescriber. The necessity and concerns scales assess positive and negative attitudes toward medication. An indication of the relative importance of these attitudes for individual patients is obtained by calculating the necessity–concerns differential. This is calculated as the difference between necessity and concerns scores, and thus had a possible range of −20 to 20. The necessity–concerns differential may be thought of as the result of a cost–benefit analysis for each patient in whom their perceptions of cost (concerns) are weighed against their perception of benefit (necessity beliefs). If the difference is positive, the patient perceives that the benefits of medication outweigh the costs. Conversely, if it is negative, the patient perceives greater cost than benefit.

d. **Expectations of treatment**: The Stanford Expectations of Treatment Scale (**SETS**) includes 6 items that are assessed using a 7 point Likert scale (strongly disagree =

1 to strongly agree = 7). The scale is scored by taking the average of items a, c and e for positive expectancy and items b, d and f are averaged for negative expectancy.

e. **Brief Illness Perception Questionnaire (B-IPQ)** (Thoughts and feelings about blood pressure): Questions are scored on a range from 0 to 10 and a total score is derived by summing the individual scores.

(vi) Intervention fidelity

a. **GP Clinical Inertia** in both groups will be assessed by analysis of treatment decisions: data regarding blood pressure measurements (clinic monitored in the control group and self-monitored in the intervention groups) in relation to target blood pressure (as defined by group at randomisation (standard hypertension, older hypertension and diabetes) will be compared to data regarding medication prescription and in particular changes in defined daily dose (DDD) .
DDD data will be calculated at baseline, 6 months and 12 months for all participants. The change is DDD will be computed between baseline and 6 months and baseline and 12 months as above. Data will be presented separately for intervention and control.
In the intervention groups, BP is measured twice a day for the first week of each month. The proportion of times the participants' weekly average BP is equal to or above the target BP (135/85mmHg for standard, 145/85 mmHg for older and 135/75 mmHg for diabetes) will be computed for each participant [e.g. baseline to 6 months: Number of weekly average BPs equal to or above target/# of weekly average BPs recorded prior to 6 month FU]. At 6 and 12 months, a graph of the proportion of BPs above target versus change in DDD will be plotted for the intervention groups to explore clinical inertia.
In the usual care (control) group, the number of times BP is recorded is variable throughout the study [recorded in CRF 6/12 months: Has the patient seen their GP or Practice Nurse at their GP surgery about their BP since their last visit]. The number of BP readings that are equal to or above the target BP (140/90mmHg for standard, 150/90mmhg for older and 140/80mmHg for diabetes) will be plotted against the change in DDD from baseline to 6/12 months.

b. **Patient Fidelity to monitoring regime** will be assessed by analysis of monitoring behaviour using data regarding blood pressure measurements including level, timing, response to high or low values. This will only be of relevance to the self-monitoring groups. These data will be obtained from data collected by the BP monitors and linked to the consultation data collected in the CRFs.

In the analysis of home recorded BP measurements, home BP readings that are

50% higher than the next highest home BP of the individual participant are considered irrelevant and discarded as were readings with SBP < 70 or >250mmHg and DBP <40 or >150mmHg) (Stergiou, AJH 1998: 11;820-27). At a minimum, for each intervention arm, we will report the median number of home BP readings per participant, the proportion of participants recording the minimum monitoring (at least 12 readings per week over 6 and 12 months) and the proportion of patients that reported a home BP outside the study limits (SBP >170 or DBP >105 or SBP <100). Additional analyses will not be reported in the primary publication and will be defined in detail elsewhere prior to analysis of the data.
The number and proportion of patients in the control arm who indicated at baseline that they had self-monitored within the last 6 months will be reported.

(vii) Lifestyle behaviour

Section C in the 12 month follow up questionnaire (Section E in baseline) collects data on alcohol, diet, exercise and smoking.

a) **Alcohol consumption** is assessed using the AUDIT-C (Alcohol Use Disorders Identification Test consumption. The scores for the three questions are summed (range 0-12) with a higher score, generally, indicating that the patient's drinking is affecting his/her safety. A score of 5 or above is considered to be AUDIT-C positive (Department of Health, 2013).

b) **Diet** is assessed using the Short Food Frequency Questionnaire which is a questionnaire with 20 foods listed and participants are requested to indicate how often these have been eaten over the past month (never to more than once a day). It also asks how many teaspoons of sugar and pints of full fat milk are used daily. A total score is computed by adding the scores for each item.

c) **Exercise** is assessed using the Godin Leisure-Time Exercise Questionnaire. A weekly activity score in metabolic equivalents (METS) is computed as = (Number of times per week strenuous exercise x 9) + (number of times per week moderate exercise x 5) + (number of times per week mild exercise x 3)

d) **Smoking** is assessed using the smoking tool kit. One question asks about smoking status and a second requests frequency data for those participants who smoke.

(viii) **Quality of life**: as measured by EQ-5D-5L scores. EQ-5D-5L index scores will be computed as per the EQ-5D-5L user guide (version 2.1 April 2015).

(ix) Resource use and costs: Resource use and costs will be analysed as part of the economic evaluation and the methods will be detailed in a separate analysis plan.

(x) Qualitative analysis:

Qualitative analyses are not covered in the scope of this statistical analysis plan. Data which fall into this category include two questions on how worried and concerned the participants are, one question on how important to the patient their blood pressure is, and the qualitative interviews undertaken from the main trial follow-up.

1.7 Sample size for primary outcome

The study requires a total sample of 1110 patients to be recruited with 370 randomised to Usual Care (UC) and 740 randomised to self-monitoring (SM) with or without telemonitoring (TM), comprising 370 in the SM only group and 370 in the SM+TM group. This is based on a common standard deviation of 17mmHg and a three way pairwise comparison (SM vs SM+, SM vs UC, SM+ vs UC), at least 367 participants per group (allowing for 15% attrition) would allow us to detect a 5mmHg difference between groups (i.e. standardised effect size = 0.3) with 90% power and an adjusted alpha of 0.017 (to account for the three way comparison). Previous experience suggests that around 120-150 practices will be required to recruit a sample of this size: assuming an average list size of 7000, with a prevalence of hypertension of 13%, of whom approximately 16% will respond to a trial invitation and 40% of these will be eligible. This corresponds to around 7-10 patients per practice.

1.8 Randomisation and blinding in the analysis stage

Eligible patients who have completed the baseline assessment were individually randomised at the baseline clinic using a web based system with manual Primary Care Clinical Trials Unit (PC-CTU) back up. Randomisation was stratified by practice and minimised on baseline BP (*SBP23*), gender, and BP target (standard hypertension, older hypertension and diabetes). Patients were randomised to one of three groups: self-monitoring alone, self-monitoring with telemonitoring, and clinic monitoring (usual care) (1:1:1). The study is not blinded but uses automated assessment of end point (SBP23).

1.9 Characteristics of participants

Baseline characteristics of the patients (i.e. demographics, duration of hypertension, past medical history, height, weight and waist circumference, blood pressure, history of CVD, current antihypertensive medication, symptoms, illness perception, anxiety, quality of life, lifestyle, adherence to medication, beliefs about medicines and expectations of treatment) will be reported by the three randomised groups.
There will be no tests of statistical significance nor confidence intervals for differences between randomised groups on any baseline variables but frequency (proportions), mean/standard deviations or median/IQR will be quoted.
Patient throughput from screening through randomisation, follow up and analysis will be presented in a CONSORT flow chart and include reasons for withdrawal (Appendix III).

1.10 Definition of population for analysis

All data will be included in the analysis as far as possible to allow full ITT analysis. Patients will be analysed in the groups to which they were allocated, irrespective of whether they received that intervention or not.

2 Data Monitoring Committee And Interim Analyses

The trial is of a method of management rather than a medicinal product and it is not anticipated that the trial will be terminated unless on the advice of the Data Monitoring Committee (DMC) in the case of a series of Suspected Unexpected Serious Adverse Reactions (SUSARs).
No interim analysis was planned in the protocol.
The planned analysis for the DMC will be detailed in a separate analysis plan.

3 Primary Analysis

The primary objective is to evaluate the management of hypertension in primary care using self monitored blood pressure, with or without telemonitoring compared to usual care.

3.1 Primary outcome

Descriptive summaries of mean blood pressure at baseline, 6 months and 12 months will be presented for each group.
A mixed effect model will be fitted to the data with systolic BP at 6 and 12 months follow-up as the dependent variable. The model will include a random intercept for each participant to account for the repeated measures on the same participant and an interaction term for the treatment by visit interaction to allow the treatment effect to differ at each visit. Included in the model will be fixed effects for randomised groups (three levels with 'usual care' as reference category), and the minimisation variables as covariates (baseline systolic BP23, gender, and BP target (standard hypertension/older hypertension/diabetes)). In addition, history of CVD will be fitted as a covariate at the recommendation of the DMC. Study site (practice) will be included in the model as a random effect. Adjusted mean

differences between randomised groups in systolic BP at 12 months with 95% confidence interval and p value will be estimated from the model for the following comparisons.

1. self-monitoring versus usual care (control),
2. self-monitoring with telemonitoring versus usual care (control)
3. Self-monitoring versus self-monitoring with telemonitoring

3.2 Handling missing data and unrealistic data

At each assessment time point, participants will have six BP measurements taken and it is possible that one (or more) of these measurements are missing or potentially erroneous. The primary analysis will use all recorded BP measurements. A sensitivity analysis will explore the impact of potentially erroneous BPs identified by a macro to identify outlying BP measurements (Appendix 3).
Where one or both *SYSBP2* and *SYSBP3* are not available, the participant will have missing data for the primary outcome at that time point. A further sensitivity analysis will include participants with one of SBP2/SBP3 measurements missing.
In the analysis accounting for habituation, If ONLY ONE reading is missing, due to machine error, the mean of four available 2-6th measurements will be used in the analysis.

The frequency (with percentage) of losses to follow-up (defaulters and withdrawals) over the 12 months of the study will be reported by randomised group and compared between the groups. Three comparisons will be made (self-monitoring vs control, telemonitoring vs control, self-monitoring vs telemonitoring). Any deaths and their causes will be reported separately.
The availability of the outcome data for the primary and secondary outcomes (blood pressure measurements only) will be summarised by the three randomised groups.
The mixed effects model implicitly accounts for data missing at random, however the data missing mechanism will be explored. A logistic regression model will explore any association between baseline characteristics and availability of the primary outcome.
Any changes to the assumptions made in the primary analysis i.e. data missing at random, will be considered in a sensitivity analysis.

3.3 Multiple comparisons and multiplicity

This is a three arm trial. The sample size used an adjusted alpha of 0.017 in order to maintain an overall Type I error rate of 5%.

3.4 Model assumptions

Model assumptions will be assessed using graphical representations of residuals.

4 Secondary Analysis

4.1 Primary outcome

To control for habituation, the mean of SBP at 12 months using measurements 2-6 will be used in the analysis. The analysis detailed for the primary outcome in section 3.1 will be repeated.

4.2 Secondary outcomes

1. Analysis of systolic BP at 6 months (mean of 2nd/3rd measurements) will be derived from the primary outcome model.

Additional blood pressure outcomes as listed below will be analysed as per the primary outcome analysis in section 3.1. Analysis of diastolic BP will adjust for baseline DBP rather than baseline SBP.

2. Systolic BP at 6 months (mean of 2nd/6th measurements)
3. Diastolic BP at 6 and 12 months (mean of the 2nd/3rd measurements) and
4. Diastolic BP at 6 and 12 months (means of 2nd/6th measurements)

4.3 Tertiary outcomes

The analyses of tertiary outcomes are listed by variable type.
Pseudo-continuous outcomes measured at 6 and 12 months (STAI, B-IPQ, current medication (DDD)) will be analysed using linear mixed effect models following a similar strategy to that outlined in the analysis of the primary outcome (section 3.1). Where available, the baseline outcome score will be included as a covariate along with minimisation variables (baseline Systolic BP, gender and BP target).
Pseudo-continuous outcomes measured 12 months (SETS (positive and negative expectancy scores), BMQ-S, , diet exercise weekly activity score, AUDIT-C,) will be analysed using linear mixed effect models. The linear mixed effect models will include site as a random effect and minimisation factors (baseline SYSBP, gender and BP target), baseline outcome variable (where applicable) and randomised group (three levels with usual care as

reference category) as fixed effects. The difference in outcome at 12 months and 95% confidence interval will be reported.
BMQ-S necessity and concerns subscales will be analysed separately as well as analysing the calculated necessity-concerns differential.
MARS is likely to be skewed therefore a generalised linear mixed model (GLMM) will be fitted to the data. The generalised linear mixed effect model will include practice as a random effect and minimisation factors (baseline SYSBP, gender and BP target), baseline MARS and randomised group (three levels with usual care as reference category) as fixed effects. The difference in outcome at 12 months and 95% confidence interval will be reported.
If the assumptions for GLMM are not satisfied, a non-parametric approach will be applied to the data and the difference in medians and 95% CI will be reported for each two group comparison.
Current medication will be analysed by drug class and overall and summarised as per the TASMIN-SR paper (table 3 below). Number of antihypertensive medications will also be analysed.

Table 3. Unadjusted Prescription of Antihypertensives (Number and Defined Daily Dose) in Intervention and Usual Care Groups[a]

	Time Point						Difference Between Intervention and Control	
	Baseline		6 Month		12 Month			
	No. of Patients	Mean (95% CI)	No. of Patients	Mean (95% CI)	No. of Patients	Mean (95% CI)	6 Month	12 Month
**No. of Antihypertensive Drugs**								
Usual care	230	1.63 (1.46 to 1.79)	226	1.75 (1.58 to 1.92)	230	1.73 (1.56 to 1.91)	0.19 (−0.01 to 0.39)	0.27 (0.07 to 0.47)
Intervention	220	1.59 (1.42 to 1.76)	215	2.07 (1.87 to 2.26)	220	2.22 (2.03 to 2.42)		
**Overall Defined Daily Dose**								
Usual care	230	2.34 (2.10 to 2.58)	226	2.57 (2.33 to 2.81)	230	2.61 (2.37 to 2.85)	0.66 (0.17 to 1.15)	0.91 (0.42 to 1.40)
Intervention	220	2.16 (1.91 to 2.40)	215	3.05 (2.80 to 3.30)	220	3.34 (3.09 to 3.59)		
**Defined Daily Dose Thiazides**								
Usual care	230	0.23 (0.17 to 0.29)	226	0.24 (0.18 to 0.30)	230	0.23 (0.17 to 0.29)	0.11 (0.02 to 0.24)	0.16 (0.04 to 0.29)
Intervention	220	0.23 (0.17 to 0.30)	215	0.35 (0.29 to 0.42)	220	0.39 (0.33 to 0.46)		
**Defined Daily Dose Calcium Channel Blockers**								
Usual care	230	0.43 (0.33 to 0.53)	226	0.52 (0.42 to 0.62)	230	0.55 (0.44 to 0.65)	0.23 (0.03 to 0.44)	0.28 (0.08 to 0.49)
Intervention	220	0.46 (0.36 to 0.57)	215	0.79 (0.68 to 0.89)	220	0.86 (0.75 to 0.96)		
**Defined Daily Dose Angiotensin-Converting Enzyme Inhibitor/Angiotensin II Receptor Blockers**								
Control		1.42 (1.24 to 1.60)	226	1.55 (1.37 to 1.73)	230	1.59 (1.41 to 1.77)	0.26 (−0.11 to 0.62)	0.34 (−0.02 to 0.70)
Intervention		1.22 (1.04 to 1.41)	215	1.61 (1.43 to 1.80)	220	1.74 (1.55 to 1.92)		
**Defined Daily Dose β-Blockers**								
Usual care	230	0.15 (0.11 to 0.19)	226	0.15 (0.11 to 0.19)	230	0.14 (0.10 to 0.18)	0.03 (−0.05 to 0.11)	0.02 (−0.06 to 0.09)
Intervention	220	0.14 (0.10 to 0.18)	215	0.17 (0.13 to 0.21	220	0.15 (0.11 to 0.19)		

[a] Defined daily dose as classified by World Health Organization. Figures combine standardized "average maintenance dose" and number of medications.[28]

Binary variables (symptoms, smoking, AUDIT-C≥5)
The number and percentages of participants experiencing an outcome will be presented by group at each time point.

Symptoms
The number and percent of people experiencing each symptom from section 6 of the CRF will be tabulated as per Table 4 of the TASMIN-SR study (McManus et. al JAMA 2014) in order from the most commonly reported to least commonly reported. All symptoms will be reported in the statistical report, not just the top 10 with separate tables at each visit (baseline, 6 months, 12 months).

Table 4. The 10 Most Frequently Reported Adverse Effects Plus Selected Hypertension Medication-Specific Symptoms or Adverse Effects at 12 Months

	No. (%) of Patients		
	Usual Care (n = 230)	Intervention (n = 220)	*P* Value
Stiff joints	110 (48)	109 (50)	.72
Pain	113 (49)	101 (46)	.49
Fatigue	106 (46)	93 (42)	.42
Swelling of legs and ankles	78 (34)	81 (37)	.52
Sleep difficulties	86 (37)	71 (32)	.26
Breathlessness	66 (29)	68 (31)	.61
Dry mouth	74 (32)	58 (26)	.18
Cough	65 (28)	64 (29)	.85
Pins and needles	61 (27)	52 (24)	.48
Loss of libido	49 (21)	48 (22)	.90
Additional hypertension medication specific symptoms			
Dizziness	43 (19	53 (24)	.16
Impotence	36 (16)	37 (17)	.74
Rash	23 (10)	18 (8)	.50

Comparisons between groups for binary outcomes measured at 6 and 12 months (i.e. symptoms, smoking, AUDIT-C ≥ 5) will be conducted using a generalised linear mixed effects model for repeated measures binary data with log-link function (log-binomial model), adjusted for baseline values (symptom experienced at baseline (Y/N). If the mixed model does not converge then separate models at 6 and 12 months will be fitted instead. If separate models do not converge then a logistic model (with logit-link function) will be utilised instead. It is unlikely that models will converge when adjusted for baseline minimisation variable so these will not be included. Separate estimates at 6 and 12 months for each group comparison (with usual care as reference category) will be derived from the model(s) and presented as adjusted relative risks with 95% confidence interval and associated p values.

Comparisons between groups for binary outcomes measured at 12 months (i.e smoking, AUDIT-C ≥ 5) will be conducted using a generalised linear mixed effects model for repeated measures binary data with log-link function (log-binomial model), adjusted for baseline values (symptom experienced at baseline (Y/N). If the mixed model does not converge then separate models at 6 and 12 months will be fitted instead. If separate models do not

converge then a logistic model (with logit-link function) will be utilised instead. It is unlikely that models will converge when adjusted for baseline minimisation variable so these will not be included. Separate estimates at 6 and 12 months for each group comparison (with usual care as reference category) will be derived from the model(s) and presented as adjusted relative risks with 95% confidence interval and associated p values.

Cardiovascular outcomes:
Cardiovascular outcomes will be summarised as counts of events per group and number (%) of participants experiencing at last one event of each type per group. Additionally, the number (%) of participants experiencing at least one event of any type (any CV event) will be summarised per group. The number of events is expected to be very small and thus analyses will not statistically compare rates of events per group.

5 Subgroup Analyses

The following subgroup analyses will be conducted with respect to the primary outcome only. These analyses should be considered exploratory. A subgroup effect will be investigated through fitting an interaction term for subgroup x randomised group x time. The results for all subgroup analyses will be reported in a forest plot, along with the overall treatment effect. In addition to the effect size and 95% CI for the treatment effect in each level of subgroup, the P value for the interaction term will be reported.

- (i) Age (two categories split at median)
- (ii) BP target (standard hypertension/older hypertension/diabetes)
- (iii) Gender (male/Female)
- (iv) Baseline BP (two categories split at median)
- (v) IMD (two categories split at median)
- (vi) History of CVD (Yes/No). History of CVD present, if the participant has any one of the following events recorded in past medical history of baseline questionnaire (angina, heart attack or MI, CABG/angioplasty/stent, stroke, peripheral vascular disease, heart failure)

6 Sensitivity analysis

Sensitivity analysis will be conducted with respect to the primary outcome only (unless explicitly stated) and will explore the sensitivity of results to different assumptions regarding missing data, outliers and departure from randomisation policy.

- (i) The mixed model assumes that the data are missing at random (MAR). A logistic regression analysis will be conducted to investigate factors (if any) that are predictive of non-response. If any factors are associated with non-response, the linear mixed effect model will be re-run with these factors included as covariates

in the model. The model will include covariate x time interaction to account for missingness over time.

(ii) Participants with a missing SBP23 measurement, will be included in the analysis with the missing SBP replaced by the average of its two immediate neighbours in the analysis of the primary outcome.

(iii) Participants with missing *SYSBP23* measurements will be imputed using multiple imputation (MI). A fully inclusive MI will be conducted with; covariates including age, height, gender, ethnicity, baseline weight and BMI, SBP and DBP measurements (1st-6th) and other factors expected to related to the main outcome (i.e. practice, diabetes status).

(iv) SBP measurements identified as outliers (Appendix 3) will be replaced by the average of its two immediate neighbours in the analysis of the primary outcome

(v) A per-protocol analysis will be conducted. In the self-monitoring arms, compliance with the protocol will be defined as 80% or more of expected blood pressure readings over 12 months [from the BP monitor data]. Compliance will be computed as total number of blood pressure readings recorded over 12 months (maximum of 4 BPs per day to be used to compute total)/ number of blood pressure readings expected over 12 months (28 x 12 = 336).

The treatment effect, its 95% confidence interval and P value will be reported under the different missing data techniques and will be compared to the treatment effect, 95%CI and P value for the primary analyses. A summary of the results will be reported by graphical methods.

7 Safety Analysis

Serious adverse events for the full population will be summarised descriptively according to randomised group. No statistical comparisons will be undertaken on these data

8 Changes to protocol or previous versions of SAP

All changes from the protocol or from previous versions of the stats plan will be detailed in the report.

1. The protocol stated that “The two self-monitoring groups will first be compared to the clinic monitoring group. If both of the treatments are found to be more effective than usual care they will be compared to each other” The sample size was adjusted to account for three comparisons, therefore all three comparison will be conducted.
.

9 Appendices

Appendix I. Schedule of study procedures

**Procedures**	**Screening/baseline**	**Follow up 1**	**Final Follow up**
Informed consent	x		
Eligibility assessment	x		
Randomisation	x		
Demographics	x		
Medical history	x	x	X
Concomitant medications	x	x	X
Adherence	x	x	X
Blood pressure	x	x	x
Height	x		
Weight	x	x	x
Trial specific questionnaires	x	x	x
Adverse event assessments		x	x

Appendix II. Outcome assessment schedule

	Baseline	6 months	12 months
Blood pressure (sitting)	x	x	x
Blood pressure (standing)	x		
Demographics	x		
Duration of hypertension	x		
Past/new medical history	x	x	x
Contraindications to anti-hypertensives	x		
Short orientation memory test	x		
Height	x		
Current antihypertensive medications	x	x	x
Weight and waist circumference	x	x	x
Symptoms part plus short form of illness perception questionnaire	x	x	x
Short form of State Trait anxiety inventory (STAI)	x	x	x
EQ-5D 5L	x	x	x
BP measurement preference	x		x
Medication Adherence Rating Scale (MARS)	x		x
Beliefs about Medicines Questionnaire	x		x
Standard Expectations of Treatment Scale (SETS)	x		x
Lifestyle questions	x		x
Loss of follow-up/withdrawal		x	x
Side effects and safety		x	x

Appendix III. Flow diagram of trial participants

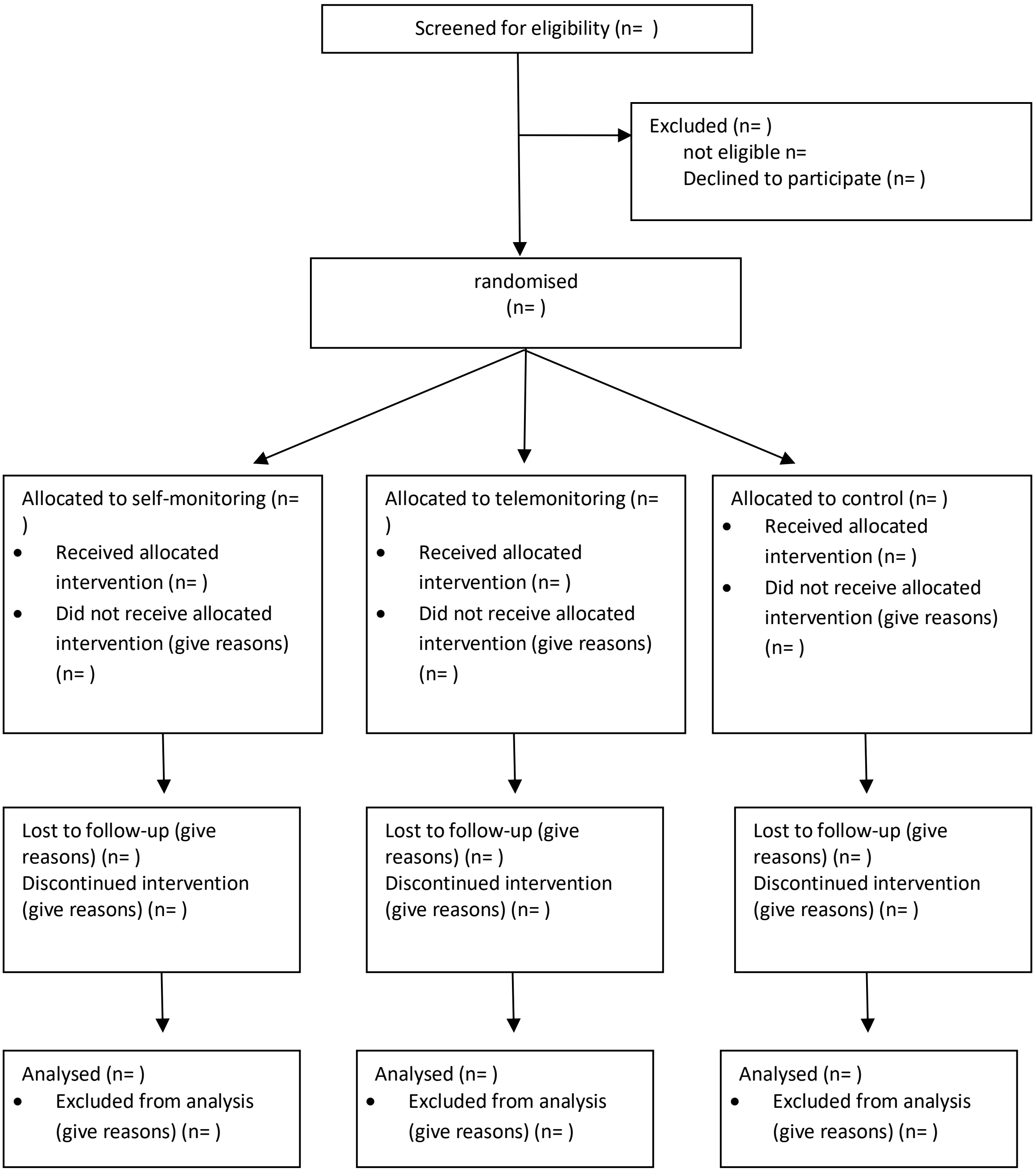

Appendix 3: Identification of outliers

Minitab Macro (BP_outlier.mac) This macro calculates the standardised difference from the mean of the smallest and the largest of the 2-6th BP readings (where the mean and the SD are calculated from the rest of the readings of the patient concerned). If the maximum of the two standardised differences is bigger than 20 then it was decided that there is an outlier. In that case the outlier is replaced by the average of its two immediate neighbours. Outlying readings are replaced by their two neighbours and then SBP23 and SBP26 measures are calculated.

```
BP_outlier.mac
gmacro
BP_outlier
Brief 0
count c2 k100
do k1=1:k100
let c10(1) = c2(k1)
let c10(1) = c3(k1)
let c10(2) = c4(k1)
let c10(3) = c5(k1)
let c10(4) = c6(k1)
let c10(5) = c7(k1)
sort c10 c11;
by c10.
if C11(5) = '*'
        let c12(1) = '*'
        let c12(2) = '*'
        let c12(3) = c11(2)
        let c12(4) = c11(3)
        let c12(5) = c11(4)
        mean c12 k3
        stdev c12 k2
        let c13(k1) = abs((c11(1) - k3)/k2)
 Else
        copy c11 c12
        let c12(1) = '*'
        stdev c12 k2
        mean c12 k3
        let c13(k1) = abs((c11(1) - k3)/k2)
Endif
sort c10 c11;
by c10;
desc c10.
if c11(1) = '*'
        let c12(1) = '*'
        let c12(2) = c11(3)
        let c12(3) = c11(4)
```

```
        let c12(4) = c11(5)
        let c12(5) = '*'
        mean c12 k3
        stdev c12 k2
        let c14(k1) = abs((c11(2) - k3)/k2)
Else
        copy c11 c12
        let c12(1) = '*'
        stdev c12 k2
        mean c12 k3
        let c14(k1) = abs((c11(1) - k3)/k2)
Endif
enddo
endmacro
```
